# Recovery in the Myogenic Program of Congenital Myotonic Dystrophy Myoblasts after Excision of the Expanded (CTG)*n* Repeat

**DOI:** 10.3390/ijms20225685

**Published:** 2019-11-13

**Authors:** Laurène M. André, Remco T.P. van Cruchten, Marieke Willemse, Karel Bezstarosti, Jeroen A.A. Demmers, Ellen L. van Agtmaal, Derick G. Wansink, Bé Wieringa

**Affiliations:** 1 Department of Cell Biology, Radboud Institute for Molecular Life Sciences, Radboud University Medical Center, 6525 GA Nijmegen, The Netherlands; Laurene.andre@radboudumc.nl (L.M.A.); marieke.willemse@radboudumc.nl (M.W.); ellenvanagtmaal@hotmail.com (E.L.v.A.); be.wieringa@radboudumc.nl (B.W.); 2 Proteomics Center, Erasmus University Medical Center, 3015 CN Rotterdam, The Netherlands; k.bezstarosti@erasmusmc.nl (K.B.);

**Keywords:** foci, genome editing, MEF2D, myoblasts, myogenesis, myotonic dystrophy, proteomics, transcriptomics, triplet repeat

## Abstract

The congenital form of myotonic dystrophy type 1 (cDM) is caused by the large-scale expansion of a (CTG•CAG)*n* repeat in *DMPK* and *DM1-AS*. The production of toxic transcripts with long trinucleotide tracts from these genes results in impairment of the myogenic differentiation capacity as cDM’s most prominent morpho-phenotypic hallmark. In the current in vitro study, we compared the early differentiation programs of isogenic cDM myoblasts with and without a (CTG)2600 repeat obtained by gene editing. We found that excision of the repeat restored the ability of cDM myoblasts to engage in myogenic fusion, preventing the ensuing myotubes from remaining immature. Although the cDM-typical epigenetic status of the DM1 locus and the expression of genes therein were not altered upon removal of the repeat, analyses at the transcriptome and proteome level revealed that early abnormalities in the temporal expression of differentiation regulators, myogenic progression markers, and alternative splicing patterns before and immediately after the onset of differentiation became normalized. Our observation that molecular and cellular features of cDM are reversible in vitro and can be corrected by repeat-directed genome editing in muscle progenitors, when already committed and poised for myogenic differentiation, is important information for the future development of gene therapy for different forms of myotonic dystrophy type 1 (DM1).

## 1. Introduction

The generation of muscle tissue during development, growth, maintenance, and ageing is a highly integrated process that generally involves the formation of distinct types of terminally differentiated myofibers [1]. These myofibers emerge after the fusion of mononucleated myoblasts into immature multinucleated myotubes. The onset of differentiation and gradual transition of muscle progenitor cells and the subsequent fusion for the formation of terminally differentiated myofibers is controlled by an intricate network of factors and machinery for the control of transcription, RNA processing and translation [2], and the turnover of gene-products [3]. Proper coordination and regulation of the underlying molecular events by helix-loop-helix (bHLH) proteins, associated myogenic regulatory transcription factors (MRFs), and RNA binding proteins is thereby highly critical [4,5,6].

Mutations in a wide range of genes, whose regulatory or structural products are essential for these cellular and molecular mechanisms that control muscle development and function, have been implicated in muscular dystrophies, characterized by the progressive degeneration and weakness of skeletal muscle [7]. Among the muscular dystrophies, congenital muscular dystrophies are a clinically and genetically heterogeneous subgroup of disorders that occur at birth or early infancy and often have a devastating course [8,9].

A typical pleiotropic and complex combination of problems in muscle maturation is seen in congenital myotonic dystrophy (cDM), the early and severely manifesting form of autosomal-dominant myotonic dystrophy type 1 (DM1; OMIM160900) [10,11,12]. cDM is characterized by the extreme expansion of an unstable (CTG)*n* trinucleotide repeat in the 3′ untranslated region of the *DM1 protein kinase* (*DMPK*) gene [13,14], resulting in repeats of over 1000 CTG triplets [15,16]. Early studies involving the histological examination of cDM muscle biopsies revealed delayed maturation and fiber immaturity [17,18]. Moreover, ex vivo analyses of primary and immortalized skeletal muscle cells taken from cDM fetuses or children with infantile myotonic dystrophy (DM) consistently showed that their myogenic capacity is significantly compromised [19,20,21].

At the molecular level, the severe disruption of developmentally regulated alternative RNA splicing and polyadenylation pathways, evoked by the expression of toxic repeat-containing transcripts from *DMPK* and its antisense gene *DM1-AS* early in myogenesis, is presumably the main cause of the muscle problems in cDM [22]. Furthermore, at the DNA level, the extreme expansion of the (CTG•CAG)*n* repeat modulates its epigenetic environment, resulting in an increased nucleosome occupancy and hypermethylation of the CpG-island surrounding the repeat [23,24,25]. Unfortunately, how these different cDM-typical abnormalities at the DNA, RNA, and protein level are mechanistically coupled to the defective myogenic capacity of cDM muscle progenitor cells has been largely unexplored.

Here, we report on a morpho-phenotypic and molecular comparison of differentiation behavior, chromatin conformation effects across the DM1 locus, and transcriptome-proteome characteristics of clonally derived, isogenic cDM myoblast lines with and without a (CTG)2600 repeat in the *DMPK* gene. This work builds on and extends our previous report, which described the process of CRISPR/Cas9-mediated editing of the DM1 locus that generated these cell lines and the immediate effects thereof on repeat fate [26]. Here, we address long-lasting consequences and describe how complete excision of the expanded repeat does not noticeably alter the cDM-specific chromatin status or transcriptional activity of alleles within the mutant DM1 locus, but does permanently modify the expression of representative muscle markers and regulatory transcription and RNA-processing factors. Furthermore, morphological aspects of differentiation are seen to be normalized during the earliest stages of the myogenic process, when myoblasts are transiting from proliferation to quiescence and subsequently fuse to become multinuclear myotubes. Therefore, cDM-specific features show distinct reversibility upon repeat excision by somatic genome editing during the stage wherein muscle cells are already committed and poised for terminal differentiation.

## 2. Results

### 2.1. Isogenic cDM Myoblasts with and without an Expanded Repeat: Use as DM1 Cell Models

To investigate how the presence of a large-scale (CTG)*n* repeat in the mutant *DMPK* allele of a cDM muscle progenitor cell (referred to as parental DM11 myoblasts) influences myoblast-to-myotube formation along the path of terminal differentiation, we generated a panel of eight isogenic myoblast lines (Figure S1A). The lines were initially generated for a study of repeat instability upon the induction of dsDNA (double strand DNA) breaks up- and downstream of the (CTG)*n* expansion by CRISPR/Cas9 genome editing [26,27].

As all myoblasts in our panel were actively cycling immortalized cells that had undergone several rounds of clonal selection and been maintained for at least seven to eight passages in vitro, we verified whether the lineages with an expanded repeat had retained nuclear foci due to abnormal protein binding and the retention of expanded *DMPK* transcripts [28]. With FISH analysis using a CAG repeat probe, on average, 4–5 *DMPK* ribonucleoprotein (RNP) foci per nucleus were detected in the parental DM11 population and in all clonal lines with the (CTG)2600 repeat (Figure S1B,C). The foci count varied between individual cells, ranging from 0 to 17 foci per nucleus. In total, 5% of the nuclei did not contain any focus. Significant foci numbers were not observed in any of the lineages without the (CTG)2600 repeat. These observations corroborate findings on earlier passages of these cells [26].

We also performed RNA FISH on five-day-old myotubes derived from the cell lines. Foci were only observed in myotubes with the (CTG)2600 repeat (Figure S2). Importantly, we observed similar variation in the foci number between nuclei within one myotube and the entire population of myotube nuclei in the culture, which provides evidence for the idea that *DMPK* expression differences between nuclei are maintained during myogenesis.

Automated immunofluorescence analysis of repeat-containing myoblasts revealed 0–15 MBNL1-positive RNP aggregates per nucleus (mean count 2–3; Figure S1D,E). These became visible as bright foci against a variable background of dispersed nuclear and cytoplasmic MBNL1 staining. MBNL1 foci were not observed in myoblasts without a repeat. The observations described here and in the previous study [29] confirm that the aberrant partitioning of MBNL family members is a persistent feature in clonally-derived cDM myoblasts with the (CTG)2600 repeat, in a manner like that seen in muscle and nerve cells from DM1 patients with long repeats [22,30]. Abnormal RNP aggregation is obviously abrogated quickly after cells have lost the ability to produce (CUG)*n* expanded RNAs from the DM1 locus.

Cell cycle analysis of growing myoblasts in adherent 2D culture, as determined by Ki-67 staining, showed that the ratio between cells in quiescence and cells that were in the active phase of the cell cycle remained similar after repeat removal (Figure S3A). Additionally, the percentage of cells in S-phase, marked by incorporating 5-ethynyl-2′-deoxyuridine (EdU) for 1 hour, did not differ between exponentially growing lines with and without the (CTG)2600 repeat (Figure S3B). These observations suggest that cell cycle regulation was largely unaltered, and that passaging before, during, and after the gene-editing procedure and presence of an expanded repeat did not overtly affect the proliferative capacity of our model cells.

### 2.2. Myogenic Fusion Is Improved after (CTG)2600 Repeat Excision

**Figure 1:**
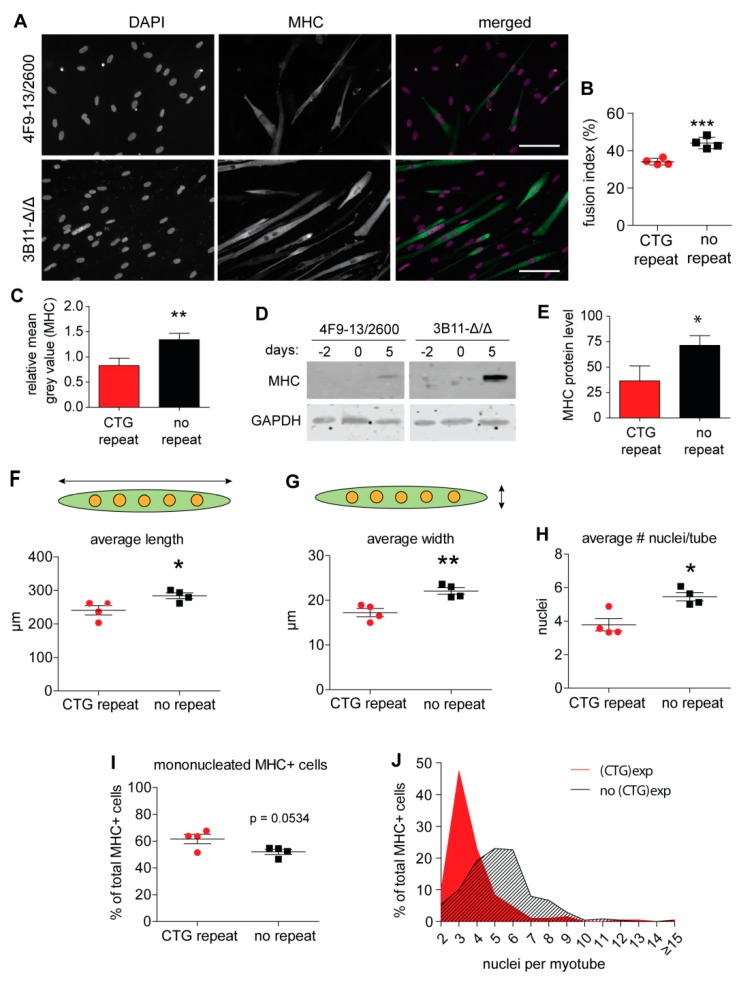
Myogenic fusion capacity of myoblasts with and without the expanded (CTG)2600 repeat. (**A**) Images of myotube formation after five days of differentiation in myoblast lineages with (4F9) and without (3B11) the (CTG)2600 repeat expansion. Myosin heavy chain (MHC) staining is shown in green, whilst DAPI is shown in magenta. Bar = 100 µm. (**B**) Fusion index: Each data point represents the mean of three independent differentiation experiments for one myoblast lineage (mean ± SEM). (**C**) Group-wise microscopic quantification of the mean grey values of MHC staining in immunofluorescent images of cells with and without a repeat. (**D**) Western blot visualization of MHC protein levels in proliferating myoblast cultures (day -2), at the start (day 0), and on day 5 of differentiation. (**E**) Quantification of MHC expression at day 5 by western blot analysis of extracts from two myoblast lineages with and two lineages without the (CTG)2600 repeat. Averaged MHC signal intensities (arbitrary units) determined from three independent differentiation experiments are shown (mean ± SEM). * *p* < 0.05, ** *p* < 0.01, *** *p* < 0.001 (two-way ANOVA). (**F**–**H**) Morphotyping of myotubes formed from myoblasts with and without the (CTG)2600 repeat. Myotubes in five-day differentiated cultures were analyzed for their average length (**F**), width (**G**), and number of nuclei per tube (**H**). (**I**) Quantification of mononucleated MHC-positive cells in the myogenic culture. (**J**) Histogram depicting the number of nuclei per myotube. Each data point in F–I represents one of the four cell lines with or without the (CTG)2600 repeat expansion, averaged for four independent experiments (mean ± SEM; on average, 56 myotubes were included per cell line per experiment). * *p* < 0.05, ** *p* < 0.01 (two-way ANOVA).

To understand the effect of (CTG)2600 repeat removal on myogenesis, we first conducted imaging and morphometric analyses to verify whether typical cDM muscle problems seen in vivo, like differentiation impairment and fiber immaturity, were lessened in the myoblasts from which the repeat was excised. As early as day 5 of differentiation, the fusion index (i.e., number of nuclei in myotubes as a percentage of the total number of nuclei in culture) was significantly higher in cell populations without than with the (CTG)2600 repeat (44% versus 34%, respectively) (Figure 1A,B). For the identification of myotubes that were formed after myoblasts were poised for fusion, we used immunofluorescent staining for myosin heavy chain (MHC) expression. Quantitative image analysis revealed that all four lines without the (CTG)2600 repeat had a significantly higher myogenic capacity than their counterparts with the repeat, resulting in a significant difference in the mean grey value for averaged MHC staining intensities between the two groups of myoblast populations (Figure 1C). Western blot imaging of MHC signals in cell extracts (Figure 1D,E) corroborated the morphological and immunofluorescence observations, confirming that MHC expression levels were significantly higher in differentiated myotubes derived from myoblasts from which the (CTG)2600 repeat was excised. When combined, these observations point to a delay in the initial period of myogenic programming in (CTG)2600 repeat-containing myoblasts, which can be relieved by repeat removal.

Morphometric tests at the single cell level confirmed our initial perception. For our morphometric analyses, we defined a myotube as an MHC-positive cell with two or more nuclei. On average, (CTG)2600 myotubes were significantly shorter and thinner and contained fewer nuclei per cell than myotubes without the repeat expansion (Figure 1F–H). Furthermore, the percentage of mononucleated MHC-positive cells—used as an indicator for myogenic cells that started aspects of the myogenic differentiation process, but did not fuse—was markedly higher in the populations of myoblasts with the repeat (Figure 1I). Based on these observations, we could conclude that, at day 5, myotubes without the (CTG)2600 tract or with a normal repeat size are morphologically more advanced and already in a more mature state than those with an expanded repeat.

The binning of all MHC-positive cells based on the average number of nuclei per cell confirmed this observation, since a conspicuous overrepresentation of mononucleated and binucleated cells was seen among the populations derived from (CTG)2600 myoblasts (Figure 1J). A few myotubes carrying the repeat expansion contained more than five nuclei, with 80% of the myotubes containing two to four nuclei. In myotubes lacking the repeat expansion, only 35% of myotubes with fewer than four nuclei were detected, indicative of the higher overall rate of myotube maturation for these lines. Apparently, the presence of the repeat slows or inhibits myogenic progression directly after the initial round of myoblast–myoblast fusion, before or early in the phase of subsequent myoblast–myotube fusion rounds. Even after 15 days of differentiation, the (CTG)2600 populations did not show a more mature myotube phenotype, indicating that the early cDM-typical fusion problems have a persistent character under our culture conditions (data not shown).

We consider it highly likely that these early myogenic abnormalities of cDM myoblasts in culture have the same underlying mechanistic cause as the well-known differentiation impairment and fiber immaturity problems seen in patient muscles in vivo [23,24]. To further deconvolute the myogenic differentiation trajectories for cells with and without the repeat, we used both informed and unbiased analyses at different time points before, at, and after the induction of the switch to quiescence and cell fusion by serum starvation, with the aim of revealing clues about pathobiological pathways that lead from DNA expansion to consequences at the transcriptome and proteome level.

### 2.3. No Change in Chromatin Status and Associated Transcriptional Activity of DM1 Locus Genes upon (CTG)2600 Repeat Excision in Myoblasts

**Figure 2:**
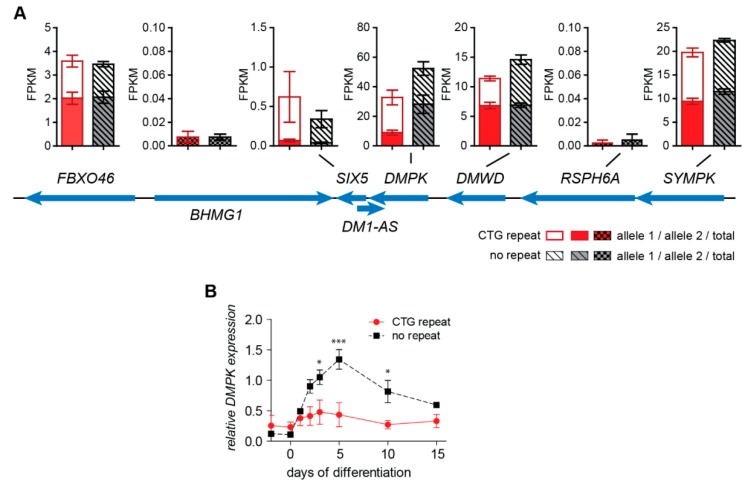
Gene expression of myotonic dystrophy type 1 (DM1) locus genes in proliferating myoblasts with and without the (CTG)2600 repeat. (**A**) Expression of the six genes flanking *DMPK/DM1-AS* (size and location on the scale depicted in blue arrows) was determined by RNA-seq analysis of poly(A)-containing RNA prepared from proliferating myoblasts with (red) and without (grey) the (CTG)2600 repeat. Based on single nucleotide polymorphism (SNP) sequence information, the contribution of the two alleles of each gene (on the DM1 chromosome or the unaffected chromosome 19) could be independently determined. It was assumed that methylation of the repeat-expanded allele decreased the expression of, e.g., *DM1 protein kinase* (*DMPK)* and *SIX5* from this allele [34]. (None of the genes featured significant differential expression levels before and after excision of the (CTG•CAG)*n* repeat.) (**B**) *DMPK* expression levels, as determined via RT-qPCR. Each data point shows the expression (mean ± SEM) in four cell lines with and without the (CTG)2600 repeat. * *p* < 0.05, *** *p*< 0.001 (two-way ANOVA).

At the level of DNA topology and function, cDM-typical alterations regarding the coding capacity of genes in the mutant DM1 locus might be involved, due to hypermethylation of the CpG island surrounding the (CTG)*n* repeat and epigenetic modification of the nearby chromatin. In a separate study focusing on methylation dynamics, we revealed that the abnormal cDM-typical heterochromatization of the locus was maintained after repeat removal in myoblasts [31]. Due to the apparent inability to change the methylation status of the DM1 locus at the somatic level, we assumed that the two sets of cell lines in our panel thus only differed in the presence of the (CTG)2600 repeat, but that they both harbor the abnormal chromatin topology of cDM cells. To investigate the consequences of this possibility in more detail and determine whether the presence of the repeat could affect the expression of neighboring DM1 locus genes in cis, we compared RNA-seq data from proliferating myoblasts with and without the (CTG)2600 repeat at 80% confluency (day -2). The use of single nucleotide polymorphism (SNP) information for the assessment of total and allelic expression of a region that covers > 150 kbp around the DM1 repeat revealed that the expression of *FBX046*, *BHMG1*, *SIX5*, *DMPK*, *DMWD*, *RSPH6A*, and *SYMPK* was not significantly different in myoblasts with and without the (CTG)2600 repeat (Figure 2A).

Importantly, whereas *DMPK* expression was essentially similar in proliferating (day -2) and confluent (day 0) myoblast cultures, significantly and persistently higher *DMPK* expression was observed in cells without the repeat during later phases of myogenic differentiation (Figure 2B). The RT-qPCR-determined profile remained relatively flat throughout the entire 15 days of myogenic differentiation for myoblasts with the repeat, whereas *DMPK* expression peaked at day 5 after the onset of differentiation in myoblasts without the repeat. The transient induction of *DMPK* mRNA production in differentiating myoblasts is a typical feature of early myogenesis and has been reported before [32,33]. To us, the near absence of a peak in the profile for (CTG)2600-containing myoblasts suggests that their normal *DMPK* expression regulation during myogenic differentiation is suppressed, presumably by an in trans effect exerted by the repeat on both alleles simultaneously. An in cis mechanism acting on the mutant allele only is less likely, as it would leave expression regulation of the normal DMPK allele unaffected; repair of the mutant allele by repeat excision in DM11 cells would then only produce a less-pronounced effect.

### 2.4. (CTG)2600-Repeat Effects on the Expression of Myogenic Transcription Factors

**Figure 3:**
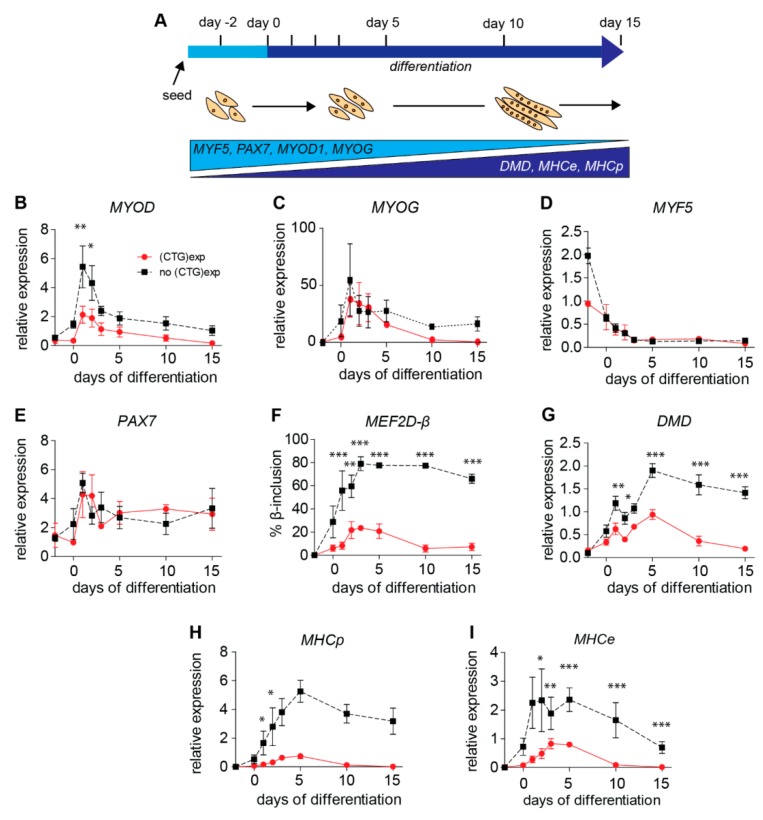
Excision of the (CTG)2600 repeat differentially alters the temporal expression of drivers and markers of myogenic differentiation. (**A**) The experimental set-up for the analysis of mRNA levels for myogenic transcription factors and structural proteins before and during myoblast–myotube differentiation in cells with and without the (CTG)2600 repeat. Time points for sampling and RT-qPCR analysis are indicated (top). Anticipated changes in the expression of muscle-specific factors are depicted (bottom). (**B**–**E**) RNA expression for myogenic transcription factors (“drivers”) *MYOD*, *MYOG*, *MYF5*, and *PAX7* (in arbitrary units). (**F**) RNA expression for the *MEF2D* + β-exon factor isoform. (**G**–**I**) RNA expression for structural muscle proteins (myogenic progression markers) *DMD, MHCp*, and *MHCe.* Each data point shows the mean ± SEM of the four different cell lines either with or without the (CTG)2600 repeat expansion. *p < 0.05, **p < 0.01, ***p < 0.001 (two-way ANOVA).

Terminal differentiation with myoblast-to-myotube transition is dominantly controlled by a network of muscle-specific transcription factors and RNA-binding proteins, involved in processing and transport. The temporal appearance and cellular expression levels of these regulatory factors (i.e., protein drivers) are under a strict regime of gene expression regulation, which, in turn, controls the production of multiple other proteins (here referred to as myogenic progression markers) that have an active or passive role in the myoblast′s transition to myotube formation and maturation (Figure 3A) [1,30,35,36].

To reveal gene-to-product expression differences in this network between cells with and without the (CTG)2600 repeat, we used RT-qPCR quantitation of transcript levels for representative transcription-driver factors *MYOD*, *MYOG*, *MYF5*, and *PAX7* at different time points over the 15-day period of myoblast growth and differentiation. Over this entire trajectory, *MYOD* expression was consistently higher in myoblasts without (CTG)2600 repeat expansion than in repeat-containing cell lines (Figure 3B). Additionally, *MYOG* levels appeared increased in lines from which the repeat was removed, but the expression was only significantly different during the second half of the test period (Figure 3C). Conspicuously, the mRNA levels for *MYF5* and *PAX7* were largely similar in both sets of cell lines (Figure 3D,E). Only early in proliferating myoblasts was *MYF5* expression two-fold higher in the lines without the repeat. Taken together, our findings indicate that the transcriptional programming of early myogenic factors is not grossly altered, but rather distorted in a subtle and selective manner by (CTG)2600 repeat presence in our cells. Importantly, repeat presence appears to have no concerted effect on the temporal expression profiles of all four regulatory factors examined.

A conspicuous exception with a more profound qualitative effect of the repeat was seen for myogenic transcription factor *MEF2D* [22,37]. RT-PCR analysis revealed that expression of the *MEF2D* mRNA variant with an included β-exon increased significantly during early differentiation (Figure 3F). In myoblasts without the repeat, the percentage of processed *MEF2D* mRNA containing the β-exon continued to increase to ~80% during the first three days of differentiation and remained constant thereafter. For the lines with the (CTG)2600 repeat, a significantly lower level of the *MEF2D* mRNA variant with β-exon (~22%) was seen, with a peak in the expression profile at day three of differentiation and a later decline.

### 2.5. Levels of Archetypal Markers of Myogenic Progression Increase after Repeat Excision

RNA expression profiling for myogenic progression markers *DMD, MHCp*, and *MHCe,* i.e., well-known structural proteins with a cytoarchitectural role whose levels are known to increase steadily during myoblast–myotube transition and the further progression of normal myogenic differentiation [1,38,39], revealed the effects of repeat removal more clearly and consistently. Throughout the entire 15-day differentiation period, the RNA expression levels of these three markers appeared significantly increased in the myoblast lines from which the repeat was excised (Figure 3G–I). A corresponding trend was observed in non-isogenic control line C25 (Figure S4).

### 2.6. (CTG)2600-Repeat Effects on the Expression of DM1 Relevant Splice Factors

The findings of others have repeatedly pointed in the direction of RNA-splicing alteration, not a change in transcriptional programming, as the main actor in impaired myogenesis by an expanded (CTG)*n* presence [22,40,41]. Earlier observations of our group regarding repeat effects on the temporal expression of the three isoforms of the *MBNL* family of RNA-binding factors, i.e., the key post-transcriptional regulators with a well-documented role in DM1 pathobiology, are concordant with this model [29]. Indeed, transcript level determination by RT-qPCR did not reveal differences in the total *MBNL1* RNA content between myoblasts with and without the repeat, but MBNL1 protein variants were significantly over- (42/43 kDa) and underexpressed (40/41 kDa) in myoblasts with the repeat. The total MBNL1 protein content was consistently two- to three-fold higher in cells without the (CTG)2600 repeat, before and after the onset of differentiation. Similarly, *MBNL2* RNA expression did not differ markedly, but the MBNL2 38/40 kDa variants were more highly expressed in repeat-containing myoblasts, whereas the MBNL2 39 kDa variant was less expressed. The level of MBNL2 was higher in myoblasts without the repeat, although this difference was most evident somewhat later during differentiation. Unfortunately, the MBNL3 protein, known to be involved in *MEF2D* splicing [42], could not be detected by western blotting, presumably due to its low expression in skeletal muscle.

For one other candidate thought to be centrally involved in the abnormal programming of RNA metabolism in DM1, CELF1, the protein expression appeared not to differ between cell lines with and without the (CTG)2600 repeat (Figure S5).

### 2.7. (CTG)2600-Repeat Removal Restores DM1-Typical Alternative Splicing Abnormalities Early in Myogenic Progression

**Figure 4:**
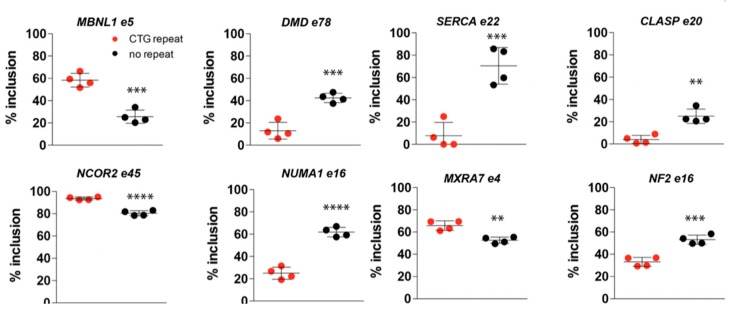
Alternative splicing patterns for early markers in myotonic dystrophy type 1 (DM1) myoblasts change from embryonic-to-adult mode after (CTG)2600 repeat removal. Analysis of RNA-seq reads across alternatively used splice junctions in transcripts from eight mis-spliced DM1-related genes in myoblasts shows the reversal of aberrant splicing after (CTG)2600 repeat removal. Each data point represents one cell line with or without the (CTG)2600 repeat. ** *p* < 0.01, *** *p* < 0.001, **** *p* < 0.0001 (two-way ANOVA).

Our findings regarding the differences in the expression of members of the *MBNL* family between cells with and without the repeat [29] support the commonly accepted model that repeat effects on the posttranscriptional regulation of RNA and protein isoform production play a dominant role in cDM manifestation in muscle. Hence, we checked whether repeat removal affected the splicing signature of known myogenic markers in proliferating myoblasts, before the onset of myogenesis. The fate of alternatively spliced exons in a number of known DM1 target transcripts was analyzed by RNA-seq. The inclusion of *DMD* e78, *SERCA1* e22, *CLASP* e20, *NCOR2* e45, *NUMA1* e16, *MXRA7* e4, and *NF2* e16 significantly differed between the populations of myoblasts with and without the (CTG)2600 repeat (Figure 4). Interestingly, for *MBNL1* e5, a clear difference was observed (Figure 4, left uppermost panel), corroborating our own and others’ earlier findings mentioned above [29,43,44,45]. Monitoring of the alternative splicing of *DMD* e78, *SERCA1* e22, *BIN1* e11, and *LDB3* e11 in differentiating myotubes confirmed that the correction of splice abnormalities was permanent and remained persistent over at least the first five days of the myogenic program (Figure S6). For all transcripts, the embryonic splice mode was shifted towards a more mature splice mode upon repeat removal, matching the situation in non-isogenic myoblast control line C25.

### 2.8. Selective Changes in the Transcriptome Composition of Myoblasts with the (CTG)2600 Repeat

**Figure 5:**
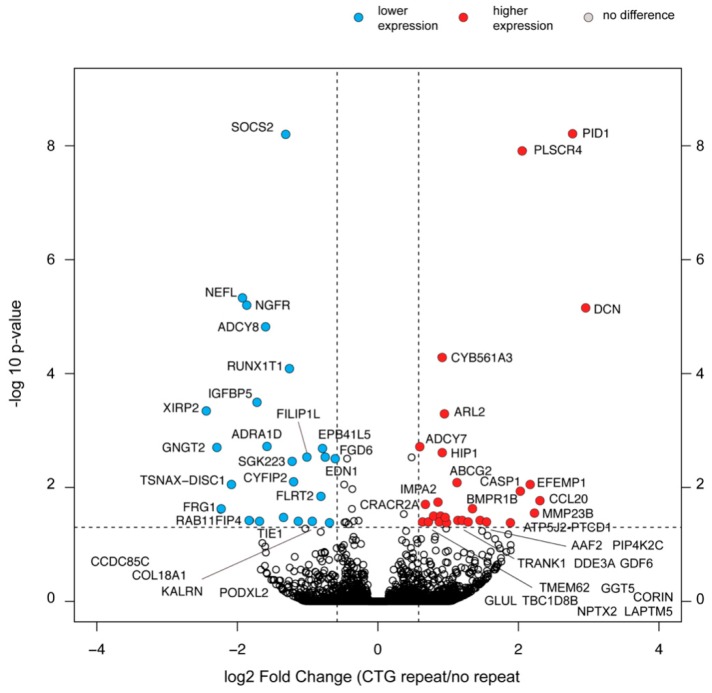
RNA-seq analysis shows few changes in RNA expression in cells with a (CTG)2600 repeat. Volcano plot showing global transcriptional changes in cells with a (CTG)2600 repeat. The log2-fold change in expression is represented on the x-axis. The y-axis shows the -log10 of the *p*-value. A *p*-value of 0.05 and a fold change of 1.5 are indicated by the black dashed lines. All 15,960 genes found in the eight cell lines are plotted. Each circle represents one gene. Twenty-four genes significantly and >1.5-fold downregulated in cell lines with the (CTG)2600 repeat are shown in blue, and 28 genes significantly and >1.5-fold upregulated in cell lines with the repeat are shown in red.

To broaden our picture of alterations triggered by expanded (CTG)*n* repeat expression, we used RNA-seq as an unbiased approach to compare the transcriptomes of the two sets of myoblast lines with and without the (CTG)2600 repeat, while still in the proliferative phase. Unexpectedly, RNA-seq analysis revealed that (CTG)2600 repeat removal caused only 52 genes, of the 15,960 genes identified, to become significantly (*p* < 0.05) differentially expressed and >1.5-fold changed (Figure 5, Table S1). Of these differentially expressed genes (DEGs), 24 were less expressed and 28 were more highly expressed in cells with a (CTG)2600 repeat. Two genes, *IGFBP5* and *AFF2* (Table S1), contain a trinucleotide repeat sequence [46].

The top four enriched Gene Ontology (GO)-pathways for the DEGs were the (i) enzyme-linked receptor protein signaling pathway (GO:0007167), (ii) regulation of cell migration (GO:0030334), (iii) cellular response to IFNγ (GO:0071346), and (iv) cAMP-mediated signaling (GO:0019933). Importantly, all four pathway-related gene sets contained one or more genes that were upregulated, as well as genes that were downregulated. We found that 21 of the 52 DEGs were previously linked to myogenesis, but none of the best known myogenic regulators of the temporal landscape of myocyte differentiation [1,36] were in this group of genes (Table S1). Adaptation in the expression and hence, the biological significance, of these 21 genes may represent a loss- or gain-of-function reaction to the presence of the repeat or be related to the gain of a more normal course of myogenesis after repeat excision. The functions of the eight genes that were more highly expressed in repeat-containing myoblasts, *LAPTM5, PDE3A, BMPR1B, CASP1, MMP23B, ABCG2, DCN*, and *GLUL*, may have the most relevant pathobiological significance and must be considered candidates for involvement in myogenic impediment in cDM1 (see Supplementary Materials for background information on genes with a known function in myogenesis). Further tests with an overexpression or knockdown of the expression of these DEGs in our myoblast lineages with the (CTG)2600 repeat are thus necessary to clarify their possible role.

As mentioned earlier, our transcriptome analysis not only revealed quantitative, but also qualitative, expression changes, related to alternative exon use (Figure 4). From the finding that our list of over- or underexpressed DEGs is relatively limited and that the inventory of alternatively spliced transcripts studied here and published by others comprises only a select group of candidates among all RNAs expressed in myoblasts, we conclude that CRISPR/Cas9-based excision of the (CTG)2600 tract has a very specific effect on the transcriptome composition. Our findings thus suggest that only a relatively small portion of early transcriptome changes upon repeat-excision may be directly associated with transcriptional effects, whereas the majority of changes must be attributed to post-transcriptional events. Secondary changes, as a result of the physiological response to the relief of cell stress caused by repeat toxicity, may also be involved.

### 2.9. Changes in the Proteome of cDM Myoblasts after (CTG)2600 Repeat Excision Are Limited and Show Little Congruence with Transcriptome Alterations

**Figure 6:**
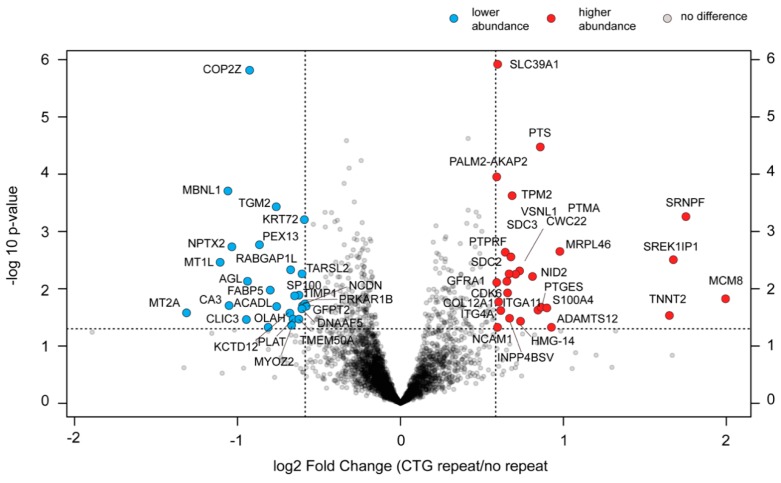
Proteomic analysis of myoblasts with and without the (CTG)2600 repeat. Volcano plot showing global changes in protein abundance in cells with a (CTG)2600 repeat. The log2 fold change is represented on the x-axis. The y-axis shows the -log10 of the *p*-value. A *p*-value of 0.05 and a fold change of 1.5 are indicated by the black dashed lines. All 5838 proteins detected in the eight cell lines are plotted. Each circle represents one protein. Proteins significantly and > 1.5-fold less abundant in cell lines with the (CTG)2600 repeat are shown in blue, and all proteins significantly and > 1.5-fold more abundant are shown in red.

Different changes in the cellular proteome, but in an equally small subset of genes, were revealed by mass spectrometry analysis. Employing an examination of the protein content of proliferating myoblasts with and without the (CTG)2600 repeat by nanoflow LC-MS/MS, we identified a total of 53 proteins, of the 5838 detected, that were significantly (*p* < 0.05) differentially expressed >1.5-fold after (CTG)2600 repeat removal (Figure 6). In total, 27 proteins were significantly more abundant and 26 proteins were less abundant in the (CTG)2600 repeat-containing lines than in the lines without the repeat (Figure 6, Table S2). The genes encoding four of them, *KCTD12*, *CDK6*, *SDC3*, and *NCAM1*, contain a trinucleotide repeat sequence [46].

Two GO-pathways were enriched for differentially expressed proteins (DEPs): (i) movement of the cell or subcellular component (GO:0006928) and (ii) cell–matrix adhesion (GO:0007160). We consider the 53 protein candidates for involvement in DM-pathobiological pathways. It is therefore important to mention that the functions of 20 proteins out of the total of 53 were previously linked to myogenesis (see Supplementary Materials). MBNL1 is within this group, which confirms our earlier finding of its underexpression in (CTG)2600 repeat-containing myoblasts using western blotting and immunofluorescence analysis [29]. Conspicuously, two members of the family of metallothionein proteins, MT1L and MT2A, were significantly less abundant in (CTG)2600 cell lines, while a third member, MT1E, did not meet the significance criterion (*p* = 0.057) to be included in the table, but actually showed the highest fold-change (log2 fold change = −1.9).

Taking these data together, we can conclude that (CTG)2600 repeat removal is associated with surprisingly few and rather selective changes in both the transcriptome and proteome signature of proliferating cDM myoblasts. Transcriptional and post-transcriptional mechanisms thereby have no congruent nature, as the effects scored by RNA-seq and MS analysis do not or hardly overlap. From this, we can conclude that the few early changes identified in proliferating myoblasts must set the stage for the more generalized phenotypical effects later, including the morphological and functional alterations after the forced induction of the differentiation program in vitro.

## 3. Discussion

Skeletal muscles are formed during prenatal development, are extensively remodeled after birth, and undergo satellite-cell-mediated regeneration upon injury throughout life [47]. From ex vivo and in vitro studies, we know that the impediment of terminal differentiation is a feature of myoblasts derived from skeletal muscles from DM1 patients who carry long expanded (CTG)*n* repeats [48,49,50]. However, details about how myogenic differentiation is impaired at the cellular and molecular level, and whether all types of muscle progenitor cells from different somitic origin in the body are affected, remain cloaked in uncertainty. Poor myogenesis [21,51,52,53], normal myogenesis with increased apoptosis [54], and normal myogenesis without increased cell death [55] have all been reported. Importantly, different cell models, including mouse C2C12 myoblasts expressing a (CTG)200 construct, MyoD-converted fibroblasts, and DM1 patient myoblasts from limb muscle origin and with different genetic backgrounds were used for these studies. Here, we are the first to report a comprehensive study on the myogenic properties of a unique series of myoblasts, which are near-isogenic and only differ in the presence of a cDM-length (CTG)2600 repeat in the DM1 locus. Our data confirm and extend the initial findings published by our own group and others on gene-edited cells in DM1 (reviewed in [27]).

Our myoblast panel was derived from gastrocnemius muscle of an 11-year old girl with the infantile form of DM1 and subjected to CRISPR/Cas9-mediated excision of *DMPK*′s (CTG)*n* segment. For interpretation of the findings presented here, we have to keep in mind that these cells originate from an *hTERT*- and *CDK4*-lentivirally immortalized population of myoblasts. Immortalization was necessary to preserve the proliferative capacity during passaging and single-cell cloning. Although it cannot be entirely excluded that the immortal phenotype may have influenced the outcome of our findings, it is of note that the groups of Furling and Mouly, from whom our DM11 cells originate, have demonstrated that the archetypal DM features of myoblasts are largely preserved [21].

Microscopic analyses confirmed (CUG)*n*-repeat appearance and MBNL1 aggregation in nuclear foci in proliferating myoblasts and in cultures of multinucleated myotubes. Foci were only seen in cells that had retained the (CTG)2600 repeat. The number of FISH-detectable foci differed between cells within a clonal myoblast population, but fell within the same range as seen for muscle cell nuclei in biopsies of DM1 patients with long repeat expansions [56] or in nuclei in MyoD-converted fibroblasts of patients [55]. The variation in foci number per nucleus can be best explained by stochastic and temporal variable initiation-elongation speeds of *DMPK* transcription in individual cells (i.e., transcriptional bursts). Similar variation was also observed for the number of MBNL1 foci, although these occurred in somewhat lower numbers. Stringent background subtraction in automated image analysis probably explains why some weaker MBNL1 foci may be missed [29].

Morphometric examination established that the DM1-typical differentiation impairment was reproduced in the (CTG)2600-containing myoblasts and evidently relieved upon excision of the repeat. We found myoblasts containing a (CTG)2600 repeat to be thinner, shorter, and to contain fewer nuclei per tube compared to their non-repeat counterparts. From our study of myoblast–myotube appearance before, at, and after differentiation induction, we inferred that repeat effects must be exerted early (day 0–3 in our set-up). Further deconvolution of the repeat effects by the use of molecular approaches corroborated this conclusion, pointing to the existence of repeat-associated problems that are already in effect before the onset of quiescence, in a phase wherein cells are poised for commitment to muscle development, but actually not yet engaged in the ensuing differentiation. Within this period and immediately thereafter, a complex program requires the activity of various protein drivers to control the production of multiple other proteins that have a role in myoblast′s transition to quiescence; metabolic change; changes in migration, adhesion, and fusion behavior; and the structural rearrangement in cytoarchitecture needed for myotube formation [1,4,5]. We can conclude that the inhibition that occurs during this first myogenic phase persists and also interferes with later myogenic programming, as more mature (CTG)2600 myotubes remained abnormal, even at day 15 of testing.

Aberrant DNA methylation of the CpG island in the DM locus does not seem to be causally involved in the atypical behavior of myoblasts with a repeat at the onset of myogenesis. Excision of the repeat in myoblasts did, unlike in cDM induced pluripotent stem cells, not lead to DNA demethylation of the CpG region to a status found in adult-onset DM1 or normal myoblasts [31]. It is therefore likely that methylation effects on chromatin configuration in the DM locus still persist in our myoblasts. Indeed, maintenance of the epigenetic status may explain why we found no significant effects from repeat excision on the allelic expression of RNAs from the DM locus genes. Further work is needed to untangle the possible relationship between the repeat presence, chromatin configuration across the DM1 locus, and myogenic differentiation capacity.

Our observations regarding the role of bHLH and MADS-domain transcription and enhancer factors in repeat-associated problems are largely in line with earlier studies. Amack and Mahadevan did not find a noticeable effect on *MyoD* and *Myf5* from (CUG)200-repeat presence in C2C12 cells, but they did report that, as in our cells, it impeded the upregulation of *MyoG* and *p21* [57]. Likewise, an altered expression of members of the MEF2 family of factors has been observed before in studies of heart and skeletal muscle tissue of DM1 patients [58,59]. Especially interesting is that the role of *MEF2D* isoform switching in activation of the myogenic program [60,61] and the process of alternative splicing are interconnected via the involvement of MBNL, and elevated MBNL3 levels are thereby correlated with impaired myogenesis via *Mef2D* β-exon exclusion [42]. Unfortunately, we could not investigate this relationship further as we were unable to detect the MBNL3 protein [29,62]. Previously, we have shown that the RNA expression of *MBNL3* was equal in myoblasts with and without a repeat, but we know from the same study that, despite the unaltered total RNA expression of family members MBNL1 and MBNL2, their protein and splice variant expression did considerably change upon repeat expression [29]. Importantly, *MBNL1* and *MBNL2* are simultaneously expressed and occur at 250- and 80-fold higher RNA levels, respectively, than the *MBNL3* in our myoblasts [29]. In sum, a more sensitive method for determining endogenous MBNL3 protein levels is required and more work is needed to resolve the initiating and prolonged effects that long repeats have on the isoform and splice variant expression and complementary roles of the MBNL family [29]. Only then can we understand the integrated function that these factors have in the coupling between the pre- and posttranscriptional networks for the control of myogenic progression in muscle [63].

Of note, the abnormal patterns of *MEF2D* variant expression and *MYOD*, *MYF5*, and *MYOG* activation in DM1 cells have a specific temporal nature [58,59]. Care must thus be taken with the precise documentation of experimental conditions and points of measurement when using the expression level of these factors as reliable early indicators for myogenic differentiation difficulties in (CTG)*n*-repeat-containing cells. Further downstream during muscle differentiation, repeat effects became more apparent, as exemplified for products of well-established marker genes for the progression of myogenesis, i.e., *DMD, MHCe,* and *MHCp*.

Multiple studies have demonstrated that RNA processing is abnormal in DM1, particularly in cDM [10,64]. We confirmed, by testing a selection of around ten transcripts, that RNA splicing differed between our two sets of myoblasts. All abnormal embryonic splice patterns were normalized in cells from which the repeat was excised. Among these, *DMD* e78 splicing is among the most widely used and generally best accepted biomarker for abnormal embryonic splicing in terminally differentiated DM1 muscle [65,66,67,68]. Our findings demonstrate that it is also a useful indicator for aberrant splice fate specification early in development, as the *DMD* gene is already expressed in committed myoblasts and binuclear myotubes. *DMD* e78 inclusion occurred 2–3 times more frequently in proliferating myoblasts lacking the repeat, well before the switch to quiescence and fusion. Altogether, we can conclude that repeat removal produces an immediate–early and lasting reversion of the RNA splicing profile, as may be expected for an abnormality whose root cause is eliminated by DNA editing. Further work must be done to prove that this fault correction also applies to alternative polyadenylation, another process that is disturbed in DM1 muscle [22,63,69].

Finally, we conducted comparative transcriptome and proteome analysis of our myoblasts to identify new annotated transcripts and protein candidates with pathobiological significance, and to generate data that can be re-evaluated when new repeat-sensitive DEG sequences become available in future DM1 studies of other cell types. The comparison of our findings with information in publicly available databases from other DM studies in tissues [22,70] may be complicated; however, as in these data sets, the superimposed effects of (i) genetic, cell type, and sample heterogeneity; (ii) neurodegenerative cell loss or senescence and the concomitant loss of cell-type specific RNAs; and (iii) autoinflammatory tissue responses and other cell-intrinsic compensatory stress responses may be reflected. We realize that in transcriptomes of myoblasts in our panel, several of the toxic effects that repeat expansion has in vivo will be missed, but the homogeneity of the cell populations allows more robust conclusions to be draw about the relevance of the distortion of gene functions, specifically in muscle progenitor cells.

Interestingly, the cellular response to IFNγ, cAMP-mediated signaling, the enzyme-linked receptor protein signaling pathway, the regulation of cell migration, the movement of cells or subcellular components, and cell–matrix adhesion were the most frequently found enriched GO-terms. An autoinflammatory disease response has been recently mentioned as a typical hallmark of neurodegenerative diseases like DM [71] and aberrant myokine and cytokine signaling activities, processes that are tightly coupled to IFNγ function, have been reported before, for both cDM muscle tissue and endocrine levels in the circulation of DM patients [72]. Similarly, cAMP/PKA signaling is required at multiple stages during myogenesis, as well as very early, in the formation of myoblasts in the myotome during embryogenesis [73]. Furthermore, the essential role of cell migration in myoblast behavior and subsequent myoblast–myotube fusion in normal muscle formation is well-recognized [74]. Additionally, on the basis of earlier findings about distortion of the cell and matrix adhesion, a possible role for cell migration in cDM pathology was anticipated [37,75]. Still, it is important to emphasize that our studies presented here suggest that a disturbed migratory capacity in DM cells may be a primary and cell-intrinsic property and feature that also must emerge at the level of 2D and 3D cell cultures in vitro. Further study of the cell migratory behavior of our myoblast lineages is necessary to confirm this idea.

Among the 52 DEGs that emerged from our RNA-seq analysis, the expression of 24 was apparently upregulated after repeat excision. In contrast, 28 types of transcripts were significantly more highly expressed in the four (CTG)2600-containing myoblast lineages than in the lineages without the repeat. We consider these DEGs and their products interesting candidates for involvement in cDM, as they may have an either active or passive role in impaired or immature myogenic regulation [76]. More research is now required to confirm and pinpoint individual activities and contributions of each of these genes in cDM.

To us, the most surprising outcome of our transcriptome and proteome analyses was that essentially no overlap was found between names in the transcriptome (DEG) and proteome (DEP) candidate lists. Only one gene, *NPTX2* (neuronal pentraxin-2), was shared. However, while the NPTX2 protein was less abundant in (CTG)2600-carrying myoblasts, *NPTX2* RNA was upregulated in these cells. This discrepancy may be explained by effects from post-transcriptional regulation or post-translational modifications and breakdown, but we do not know whether this observation has any pathobiological significance. Additionally, the fact that *NPTX2*′s role has, until now, only been connected to synapse formation, not myogenesis, does not help to reveal clues [77]. For now, more relevant may be that the roles of MT2A, MBNL1, CA3, AGL, FABP5, NID2, ITGA11, S100A4, and TNNT2 have already been linked to events in myogenesis before (Table S2 and Supplementary Materials). The finding that MBNL1 was among the proteins downregulated by repeat presence confirms our own observations obtained using western blotting [29] and those of many colleagues in the field, and supports the key role of this RNA-processing factor in DM.

Of note, some of the protein candidates that were differentially overexpressed in repeat-containing myoblasts have an annotated role in cell–matrix adhesion, like NID2, ITGA11, and ADAMTS12, suggesting that alterations in cell–matrix adhesion are an important feature. This confirms the GO-term enrichment findings from our RNAseq study and supports the results published by Batra et al., obtained from an “omics” study of DM1 mouse models [78]. Finally, no less than three members of the metallothionein (MT) family, MT1E, MT1L, and MT2A, were among the clearly underexpressed proteins in (CTG)2600 myoblasts. MTs are proteins without a yet clearly defined physiological role, whose expression is induced in cells when put under stress conditions, e.g., by zinc, glucocorticoids, or oxidative stress. MT expression in skeletal muscle tissue was reported to be elevated under conditions of atrophy [79], in sarcopenic muscle or under specific conditions of muscle immobilization [80], rat muscle atrophy from different causes [81], or in vitro upon induced atrophy in C2C12 cells [82]. Blocking MT1 and MT2 levels resulted in an increased myotube size in vitro and increased muscle strength in vivo in MT null mice. Our finding is thus in contrast to expectations, and does not support a direct role of MT proteins in the reduction of myotube size and fusion capacity of the (CTG)2600 cell lines in our panel. Moreover, the downregulation of MTs, instead of activation, as would be expected in response to general repeat-induced stress conditions, cannot be easily explained. Still, the fact that three MT family members appear in parallel in the list makes them interesting candidates for a further survey of their role in coping with (CTG)-repeat toxicity in muscle cells.

In conclusion, our work in a newly developed myoblast panel has confirmed several well-established ideas about cDM muscle pathobiology. The production of toxic transcripts with long triplet repeat tracts has in trans effects on the normal physiological role of other RNAs in the development, growth, and regeneration of muscle in cDM patients. These effects result in impairment of the myogenic differentiation capacity as the most prominent morpho-phenotypic hallmark. We believe that our observations generate more detail for this scenario. The specific repeat effects on muscle regulatory and marker proteins appear as distinct events in the temporal landscape of myocyte differentiation. This is most easily explained by assuming that early induced differentiation impairment acts selectively, only affecting distinct branches of the myogenic program. In such a model, repeat toxicity would be the upstream effector for particular cellular abnormalities, but leave other aspects of cellular commitment and muscle differentiation unaffected. We consider our finding—that molecular and cellular features of cDM can be reversed through gene editing in myogenic progenitor cells—important information for the development of gene therapy for skeletal muscle in DM1.

## 4. Materials and Methods

### 4.1. Cell Culture

Immortalized human DM11 myoblasts with/without (CTG)13 and/or the(CTG)2600 repeat [26] and immortalized human C25 healthy control myoblasts (CTG5/CTG14) were propagated in a 1:1 mix of Skeletal Muscle Cell Growth Medium (PromoCell; Heidelberg, Germany) and F-10 Nutrient mix (Gibco; Carlsbad, CA, USA), supplemented with 15% (*v*/*v*) Hyclone fetal bovine serum (GE Healthcare) and glutamax (Gibco). Cells were grown in adherent culture on dishes coated with 0.1% gelatin (Sigma-Aldrich). For the differentiation of myoblasts to myotubes, cells were grown to confluency until their alignment was confirmed visually. Then, proliferation medium was replaced by differentiation medium containing DMEM supplemented with 1% glutamax, 10 μg/mL insulin (Sigma-Aldrich; St. Louis, MO, USA), and 100 μg/mL apo-transferrin (Sigma-Aldrich). These low-serum conditions were maintained for the number of days indicated in the various experiments, and the medium was changed every other day. All myoblasts and myotubes were cultured at 7.5% CO_2_ and 37 °C.

### 4.2. EdU and Ki-67 Proliferation Assays

Myoblasts were grown on 0.1% gelatin-coated coverslips until 70% confluency over 48 h and incubated for 1 h in culture medium containing 20 μM EdU (5-ethynyl-2′-deoxyuridine; Thermo Fisher Scientific). Adherent cells were fixed in 2% paraformaldehyde in 0.1 M phosphate buffer for 15 min at room temperature (RT); washed three times with phosphate-buffered saline (PBS); and permeabilized with blocking buffer containing 0.1% Triton-X100 (Sigma-Aldrich), 0.1% glycin (Merck), and 3% BSA (Sigma-Aldrich) in PBS for 30 min at RT. After incubation for 3 h with anti-Ki-67 antibody (Sp6, Thermo Fisher Scientific; Boston, MA, USA) in blocking buffer, samples were washed three times with PBS and incubated with goat-anti-rabbit AF568 (Thermo Fisher Scientific) in blocking buffer for one hour at room temperature. EdU was visualized using the click-iT EdU imaging kit (Thermo Fisher Scientific), as per the manufacturers’ instruction. Coverslips were mounted on microscope slides in DAPI-containing Mowiol and images were collected on a Leica DMI6000B microscope with a 63× objective. DAPI, Ki-67, and EdU positive nuclei were counted automatically using FIJI software (v2.0).

### 4.3. RNA Isolation and RT-qPCR

RNA was isolated using the Aurum Total RNA Mini Kit (Bio-Rad; Hercules, CA, USA) and the total RNA yield per sample was determined by absorbance at 260/280 nm (NanoVUE spectrophotometer, GE Healthcare Life Sciences; Chalfont St. Giles, UK). RNA was reverse transcribed using the iScript™ cDNA Synthesis Kit (Bio-Rad). For quantitative PCR (qPCR), 3 µL 10-fold diluted cDNA preparation was mixed in a final volume of 10 µL containing 5 µL iQ™ SYBR^®^ Green Supermix (Bio-Rad) and 4 pmol of each primer (primer sequences are listed in Table S3). Samples were analyzed using a CFX96 Real-time System (Bio-Rad). A melting curve was obtained for each sample in order to confirm single product amplification. cDNA samples from the no template control (NTC) and no reverse transcriptase control (NRT) were included as negative controls. RT-qPCR quantitation of expression levels of *GAPDH* and *HPRT1* was used for normalization.

### 4.4. Validation of Alternative Splicing by RT-PCR

To analyze the splicing for *BIN1* e11, *DMD* e78, *SERCA1* e22, and *LDB3* e11, a PCR was performed with the primers listed in Table S3 using Q5 high-fidelity DNA polymerase (Bio-Rad). The program involved initial denaturation at 98 °C for 3 min, followed by 30 cycles consisting of the following steps: 98 °C for 10 s, the indicated annealing temperature for 30 s, and 72 °C for 30 s. Additionally, a final extension at 72 °C for 10 min was performed. Samples from NTC and NRT were included as negative controls. The percentage of exon inclusion was determined after quantification of the embryonic and adult splice variant on agarose gel with ImageJ software. For the β-exon inclusion of *MEF2D*, PCR samples were run on a QIAxcel Advanced capillary electrophoresis apparatus (Qiagen) and analyzed using the accompanying QIAxcel screengel software.

### 4.5. Myogenic Fusion Index Determination and Myotube Characterization

The myogenic fusion index was determined by growing and differentiating myoblasts in adherent culture in 0.1% gelatin-coated IBIDI 8-wells, as described above. Cells were fixed at different time points in 2% paraformaldehyde in 0.1 M phosphate buffer for 15 min at RT. After fixation, cells were washed three times with PBS and permeabilized with blocking buffer containing 0.1% Triton-X100 (Sigma-Aldrich), 0.1% glycin (Merck), and 3% BSA (Sigma-Aldrich) in PBS for 30 min at room temperature. After overnight incubation at 4 °C with anti-MHC antibody MF-20 (DSHB; Iowa City, IA, USA) in blocking buffer, the samples were washed three times with PBS and incubated with goat-anti-mouse AF488 (Thermo Fisher Scientific) and 100 ng/mL DAPI (Sigma-Aldrich) in blocking buffer for one hour at room temperature. After three PBS washes, samples were stored in PBS at 4 °C until imaging using a Leica DMI6000B microscope with a 20× objective. The fusion index was calculated by determining the number of nuclei in an MHC-positive area divided by the total number of nuclei present in the imaging area, which was done using ImageJ software. A detailed analysis of myotube length and width was conducted manually with FIJI software. The number of nuclei per myotube was counted and scored by hand. Two researchers independently analyzed four differentiation experiments for all eight cell lines, while sample identity was blinded. Averages of the two scorings were used for further statistical analysis.

### 4.6. RNA Fluorescence In Situ Hybridization (FISH) and Image Analysis of RNP Foci

DM11 myoblasts were grown on 0.1% gelatin-coated glass cover slips to 50–60% confluency. The cells were washed once with PBS and fixed in 4% formaldehyde and 5 mM MgCl_2_ in PBS for 10 min at room temperature. The coverslips were washed three times for 5 min with PBS and incubated in 70% ice-cold ethanol overnight. After refreshing the 70% ethanol, the fixed cell-containing coverslips were washed twice in PBS at room temperature. Coverslips were prehybridized in 40% deionized formamide (Ambion; Foster City, CA, USA) in 2×SSC (Ambion) for 20 min at room temperature, followed by overnight hybridization at 37 °C with an 0.1 ng/μL LNA-(CAG)6-TYE563 probe (Exiqon, Vedbæk, Denmark) in hybridization buffer containing 40% deionized formamide, 2 mg/mL BSA (Sigma-Aldrich), 100 mg/mL dextran sulfate (Pharmacia), 0.1% Triton X-100 (Sigma-Aldrich), 1 mg/mL herring sperm DNA (Promega; Madison, WI), 100 μg/mL yeast tRNA (Ambion), 2 mM vanadyl ribonucleoside complex (NEB; Ipswich, MA, USA), and 2×SSC. Coverslips were washed two times for 5 min with PBS before the staining of cell nuclei with 100 ng/mL DAPI (Sigma) in PBS for 10 min at RT. Coverslips were then washed twice for 5 min with PBS and mounted with Mowiol Fluorescent images that were acquired using a Leica DMI6000B microscope with a 63× objective, in three different wavelength intervals using filter sets for DAPI, FITC, and TRITC/CyImages, which were subsequently analyzed using ImageJ software. DAPI masks were created using auto-thresholding by employing Huang′s method [83], followed by a watershed. For analysis, a Find Maxima option in FIJI was applied for the TRITC channel using a noise tolerance of 200, resulting in images containing single points. Positive pixels were counted in the nucleus using the previously made DAPI masks.

### 4.7. Protein Extraction and Western Blotting

Protein of proliferating myoblasts or differentiating myotubes was extracted after two PBS washes in 2× Laemmli sample buffer and denatured by boiling for 5 min at 95 °C. Samples were electrophoresed through 8%, 10%, or 15% SDS polyacrylamide gels in SDS-containing running buffer. Proteins were transferred to Immobilon PVDF membrane (GE Healthcare, 0.45 µm pore size) and membranes were blocked for one hour with 5% skim milk powder in Tris-buffered saline with 0.1% Tween-20 (TBST) or PBS with 0.1% Tween-20 (PBST). Blots were then incubated with primary antibodies (anti-MHC; anti-MBNL1, DSHB) diluted in blocking buffer overnight at 4 °C. Membranes were washed three times in TBST or PBST and incubated with appropriate IRDeye secondary antibody diluted 1: 10,000 in TBST or PBST for one hour and washed three times before being scanned in 700 nm and 800 nm wavelength channels on the Odyssey Clx imaging system (LI-COR Biosciences; Lincoln, Nebraska USA). Densitometry was performed using Image studio version 5.0 software (LI-COR Biosciences).

### 4.8. RNA-Sequencing

RNA sequencing data from the myoblasts, proliferating at 80% confluency, were previously deposited in Gene Expression Omnibus under accession code GSE127296 [29]. Splice variants and the quantification of transcript levels of DM1 locus genes were re-analysed here in a similar fashion as described in [29]. In short, for the analysis of alternative splicing, rMATS v3.0.9 [84] was used and for quantification of the total expression levels, RSEM v1.2.12 [85] was used after merging reads for transcripts with an identical reference transcript with BowTie2 v2.2.5 [86]. To estimate allele-specific expression levels based on single nucleotide polymorphisms (SNPs), the relative occurrence was quantified using Integrative Genomics Viewer [87] and averaged to gain an estimation of each gene for the following SNPs in dbSNP [88]: rs11537711 (*FBXO56*), rs2014377, rs2014576 (*SIX5*), rs672348, rs522769, rs659444 (*DMPK*), rs2070736, rs617988, rs8110017 (*DMWD*), and rs17850110 (*SYMPK*). R-studio version 1.2.1335 was used for generating heatmaps and volcano plots of the data. Gene Ontology (GO)-enrichment analysis was performed using the GO-enrichment analysis and visualization tool (Gorilla) [35,89].

### 4.9. Protein Preparation, Mass Spectrometry, and Data Analysis

Protein from proliferating myoblasts growing at 80% confluency was isolated and on-bead digested with trypsin. Extracted proteolytic peptides were labeled with TMT 8-plex labeling reagents (Thermo Scientific), allowing for peptide quantitation. Peptides were mixed at the 8-plex level and further fractionated by HILIC chromatography. Fractions were collected and analyzed by nanoflow LC-MS/MS. nLC-MS/MS was performed on EASY-nLC 1200 coupled to an Orbitrap Lumos Tribid mass spectrometer (Thermo Scientific) operating in positive mode and equipped with a nanospray source. Peptides were separated on a ReproSil C18 reversed phase column (Dr Maisch GmbH; column dimensions 15 cm × 50 µm, packed in-house) using a linear gradient from 0% to 80% B (A = 0.1 % formic acid; B = 80% (*v*/*v*) acetonitrile, 0.1 % formic acid) for 70 min and at a constant flow rate of 200 nL/min using a splitter. The column eluent was directly sprayed into the ESI source of the mass spectrometer. Mass spectra were acquired in continuum mode; fragmentation of the peptides was performed in data-dependent mode using the multinotch SPS MS3 reporter ion-based quantification method.

Data were analyzed with Proteome Discoverer Peak lists were automatically created from raw data files using the Mascot Distiller software (version 2.3; MatrixScience). The Mascot search algorithm (version 2.3.2, MatrixScience) was used for searching against the Uniprot database (taxonomy: *Homo sapiens*, version July 2016). The peptide tolerance was typically set to 10 ppm and the fragment ion tolerance was set to 0.8 Da. A maximum number of two missed cleavages by trypsin was allowed and carbamidomethylated cysteine and oxidized methionine were set as fixed and variable modifications, respectively. The target false disovery rate (FDR) for both peptide and protein validation was set to 1%. Typical contaminants were omitted from the output tables. MetaboAnalyst (https://www.metaboanalyst.ca) version 4.0 was used for generating heatmaps and volcano plots of the data [90]. Gene Ontology (GO)-enrichment analysis was performed using the GO-enrichment analysis and visualization tool (Gorilla) [35,89]. The mass spectrometry proteomics data have been deposited in the ProteomeXchange Consortium via the PRIDE partner repository with the dataset identifier PXD016056.

### 4.10. Statistical Analysis

All experiments were performed in triplicate unless otherwise specified and representative results are shown. Statistical analysis was performed using Prism software (4.01; GraphPad, LaJolla, CA, USA), using a two-way ANOVA or one-way ANOVA, as indicated in figure legends, with α = 0.05. * *p* < 0.05, ** *p* < 0.01, *** *p* < 0.001, and **** *p* < 0.0001.

## Supplementary Materials

Supplementary materials can be found at https://www.mdpi.com/1422-0067/20/22/5685/s1.

## References

[1] Bentzinger C.F., Wang Y.X., Rudnicki M.A., Bentzinger C.F., Wang Y.X., Rudnicki M.A. (2012). Building muscle: Molecular regulation of myogenesis. Cold Spring Harb. Perspect. Biol..

[2] Tierney M.T., Sacco A. (2016). Satellite Cell Heterogeneity in Skeletal Muscle Homeostasis. Trends Cell Biol..

[3] Bell R.A.V., Al-Khalaf M., Megeney L.A. (2016). The beneficial role of proteolysis in skeletal muscle growth and stress adaptation. Skelet. Muscle.

[4] Bassel-Duby R., Olson E.N. (2006). Signaling Pathways in Skeletal Muscle Remodeling. Annu. Rev. Biochem..

[5] Buckingham M., Rigby P.W.J. (2014). Gene Regulatory Networks and Transcriptional Mechanisms that Control Myogenesis. Dev. Cell.

[6] Hentze M.W., Castello A., Schwarzl T., Preiss T. (2018). A brave new world of RNA-binding proteins. Nat. Rev. Mol. Cell Biol..

[7] Shin J., Tajrishi M.M., Ogura Y., Kumar A. (2013). Wasting mechanisms in muscular dystrophy. Int. J. Biochem. Cell Biol..

[8] Guiraud S., Aartsma-Rus A., Vieira N.M., Davies K.E., van Ommen G.-J.B., Kunkel L.M. (2015). The Pathogenesis and Therapy of Muscular Dystrophies. Annu Rev. Genom. Hum. Genet..

[9] Rahimov F., Kunkel L.M. (2013). The cell biology of disease: Cellular and molecular mechanisms underlying muscular dystrophy. J. Cell Biol..

[10] André L., Ausems C.R.M., Wansink D.G., Wieringa B. (2018). Abnormalities in Skeletal Muscle Myogenesis, Growth and Regeneration in Myotonic Dystrophy. Front. Neurol..

[11] Meola G., Cardani R. (2015). Myotonic Dystrophy Type 2: An Update on Clinical Aspects, Genetic and Pathomolecular Mechanism. J. Neuromuscul. Dis..

[12] Thornton C., Griggs R., Moxley R. (1994). Myotonic dystrophy with no trinucleotide repeat expansion. Ann. Neurol..

[13] Mahadevan M., Tsilfidis C., Sabourin L., Shutler G., Amemiya C., Jansen G., Neville C., Narang M., Barcelo J., O’Hoy K. (1992). Myotonic dystrophy mutation: an unstable CTG repeat in the 3’ untranslated region of the gene. Science.

[14] Brook J.D., McCurrach M.E., Harley H.G., Buckler A.J., Church D., Aburatani H., Hunter K., Stanton V.P., Thirion J.P., Hudson T. (1992). Molecular basis of myotonic dystrophy: Expansion of a trinucleotide (CTG) repeat at the 3′ end of a transcript encoding a protein kinase family member. Cell.

[15] Arsenault M., Prévost C., Lescault A., Laberge C., Puymirat J., Mathieu J. (2006). Clinical characteristics of myotonic dystrophy type 1 patients with small CTG expansions. Neurology.

[16] Ho G. (2015). Congenital and childhood myotonic dystrophy: Current aspects of disease and future directions. World J. Clin. Pediatrics.

[17] Farkas-Bargeton E., Barbet J.P., Dancea S., Wehrle R., Checouri A., Dulac O. (1988). Immaturity of muscle fibers in the congenital form of myotonic dystrophy: its consequences and its origin. J. Neurol. Sci..

[18] Furling D., Lemieux D., Taneja K., Puymirat J. (2001). Decreased levels of myotonic dystrophy protein kinase (DMPK) and delayed differentiation in human myotonic dystrophy myoblasts. Neuromuscul. Disord..

[19] Pelletier R., Hamel F., Beaulieu D., Patry L., Haineault C., Tarnopolsky M., Schoser B., Puymirat J. (2009). Absence of a differentiation defect in muscle satellite cells from DM2 patients. Neurobiol. Dis..

[20] Mamchaoui K., Trollet C., Bigot A., Negroni E., Chaouch S., Wolff A., Kandalla P.K., Marie S., Di Santo J., St Guily J.L. (2011). Immortalized pathological human myoblasts: Towards a universal tool for the study of neuromuscular disorders. Skelet. Muscle.

[21] Arandel L., Polay Espinoza M., Matloka M., Bazinet A., De Dea Diniz D., Naouar N., Rau F., Jollet A., Edom-Vovard F., Mamchaoui K. (2017). Immortalized human myotonic dystrophy muscle cell lines to assess therapeutic compounds. Dis. Models Mech..

[22] Thomas J.D., Sznajder Ł.J., Bardhi O., Aslam F.N., Anastasiadis Z.P., Scotti M.M., Nishino I., Nakamori M., Wang E.T., Swanson M.S. (2017). Disrupted prenatal RNA processing and myogenesis in congenital myotonic dystrophy. Genes Dev..

[23] Barbé L., Lanni S., López-Castel A., Franck S., Spits C., Keymolen K., Seneca S., Tomé S., Miron I., Letourneau J. (2017). CpG Methylation, a Parent-of-Origin Effect for Maternal-Biased Transmission of Congenital Myotonic Dystrophy. Am. J. Hum. Genet..

[24] Yanovsky-Dagan S., Avitzour M., Altarescu G., Renbaum P., Eldar-Geva T., Schonberger O., Mitrani-Rosenbaum S., Levy-Lahad E., Birnbaum R.Y., Gepstein L. (2015). Uncovering the Role of Hypermethylation by CTG Expansion in Myotonic Dystrophy Type 1 Using Mutant Human Embryonic Stem Cells. Stem Cell Rep..

[25] Buckley L., Lacey M., Ehrlich M. (2016). Epigenetics of the myotonic dystrophy-associated *DMPK* gene neighborhood. Epigenomics.

[26] Van Agtmaal E.L., André L.M., Willemse M., Cumming S., van Kessel I.D.G., van den Broek W.J.A.A., Gourdon G., Furling D., Mouly V., Monckton D.G. (2017). CRISPR/Cas9- induced (CTG•CAG)n repeat instability in the myotonic dystrophy type 1 locus: implications for therapeutic genome editing. Mol. Ther..

[27] Raaijmakers R.H.L., Ripken L., Ausems C.R.M., Wansink D.G. (2019). CRISPR/Cas Applications in Myotonic Dystrophy: Expanding Opportunities. Int. J. Mol. Sci..

[28] Mankodi A., Urbinati C.R., Yuan Q.P., Moxley R.T., Sansone V., Krym M., Henderson D., Schalling M., Swanson M.S., Thornton C.A. (2001). Muscleblind localizes to nuclear foci of aberrant RNA in myotonic dystrophy types 1 and 2. Hum. Mol. Genet..

[29] André L., van Cruchten R., Willemse M., Wansink D. (2019). (CTG) n repeat-mediated dysregulation of MBNL1 and MBNL2 expression during myogenesis in DM1 occurs already at the myoblast stage. PLoS ONE.

[30] Meola G., Cardani R. (2015). Myotonic dystrophies: An update on clinical aspects, genetic, pathology, and molecular pathomechanisms. Biochim. Et Biophys. Acta-Mol. Basis Dis..

[31] Yanovsky-Dagan S., Bnaya E., Diab M.A., Handal T., Zahdeh F., van den Broek W.J.A.A., Epsztejn-Litman S., Wansink D.G., Eiges R. (2019). Deletion of the CTG Expansion in Myotonic Dystrophy Type 1 Reverses DMPK Aberrant Methylation in Human Embryonic Stem Cells but not Affected Myoblasts. bioRxiv.

[32] Gudde A.E.E.G.E.G., González-Barriga A., van den Broek W.J.A.A.A.A., Wieringa B., Wansink D.G. (2016). A low absolute number of expanded transcripts is involved in myotonic dystrophy type 1 manifestation in muscle. Hum. Mol. Genet..

[33] Poulos M.G., Batra R., Li M., Yuan Y., Zhang C., Darnell R.B., Swanson M.S. (2013). Progressive impairment of muscle regeneration in muscleblind-like 3 isoform knockout mice. Hum. Mol Genet..

[34] Brouwer J.R., Huguet A., Nicole A., Munnich A., Gourdon G. (2013). Transcriptionally Repressive Chromatin Remodelling and CpG Methylation in the Presence of Expanded CTG-Repeats at the DM1 Locus. J. Nucleic Acids.

[35] Eden E., Lipson D., Yogev S., Yakhini Z. (2007). Discovering motifs in ranked lists of DNA sequences. PLoS Comput. Biol..

[36] Thomas J.D., Oliveira R., Sznajder Ł.J., Swanson M.S. (2018). Myotonic Dystrophy and Developmental Regulation of RNA Processing. Compr. Physiol..

[37] Bland C.S., Wang E.T., Vu A., David M.P., Castle J.C., Johnson J.M., Burge C.B., Cooper T.A. (2010). Global regulation of alternative splicing during myogenic differentiation. Nucleic Acids Res..

[38] Buj-Bello A., Furling D., Tronchere H., Laporte J., Lerouge T., Butler-Browne G.S., Mandel J.L. (2002). Muscle-specific alternative splicing of myotubularin-related 1 gene is impaired in DM1 muscle cells. Hum. Mol. Genet..

[39] Bryson-Richardson R.J., Currie P.D. (2008). The genetics of vertebrate myogenesis. Nat. Rev. Genet..

[40] Osborne R.J., Thornton C.A. (2006). RNA-dominant diseases. Hum. Mol Genet..

[41] Ranum L.P.W., Cooper T.A. (2006). Rna-Mediated Neuromuscular Disorders. Annu. Rev. Neurosci..

[42] Lee K.S., Cao Y., Witwicka H.E., Tom S., Tapscott S.J., Wang E.H. (2010). RNA-binding protein muscleblind-like 3 (MBNL3) disrupts myocyte enhancer factor 2 (Mef2) β-exon splicing. J. Biol. Chem..

[43] Lin X., Miller J.W., Mankodi A., Kanadia R.N., Yuan Y., Moxley R.T., Swanson M.S., Thornton C.A. (2006). Failure of MBNL1-dependent post-natal splicing transitions in myotonic dystrophy. Hum. Mol. Genet..

[44] Botta A., Malena A., Tibaldi E., Rocchi L., Loro E., Pena E., Cenci L., Ambrosi E., Bellocchi M.C., Pagano M.A. (2013). *MBNL1_42_* and *MBNL1_43_* gene isoforms, overexpressed in DM1-patient muscle, encode for nuclear proteins interacting with Src family kinases. Cell Death Dis..

[45] Dhaenens C.M., Schraen-Maschke S., Tran H., Vingtdeux V., Ghanem D., Leroy O., Delplanque J., Vanbrussel E., Delacourte A., Vermersch P. (2008). Overexpression of MBNL1 fetal isoforms and modified splicing of Tau in the DM1 brain: Two individual consequences of CUG trinucleotide repeats. Exp. Neurol..

[46] Kozlowski P., de Mezer M., Krzyzosiak W.J. (2010). Trinucleotide repeats in human genome and exome. Nucleic Acids Res..

[47] Dumont N.A., Bentzinger C.F., Sincennes M.-C., Rudnicki M.A. (2015). Satellite Cells and Skeletal Muscle Regeneration. Compr. Physiol..

[48] Timchenko N.A., Iakova P., Cai Z., James R., Timchenko L.T., Smith J.R. (2001). Molecular Basis for Impaired Muscle Differentiation in Myotonic Dystrophy Molecular Basis for Impaired Muscle Differentiation in Myotonic Dystrophy. Mol. Cell. Biol..

[49] Thornell L.-E., Lindstöm M., Renault V., Klein A., Mouly V., Ansved T., Butler-Browne G., Furling D. (2009). Satellite cell dysfunction contributes to the progressive muscle atrophy in myotonic dystrophy type. Neuropathol. Appl. Neurobiol..

[50] Renna L.V., Cardani R., Botta A., Rossi G., Fossati B., Costa E., Meola G. (2014). Premature senescence in primary muscle cultures of myotonic dystrophy type 2 is not associated with p16 induction. Eur. J. Histochem..

[51] Amack J.D., Reagan S.R., Mahadevan M.S. (2002). Mutant DMPK 3′-UTR transcripts disrupt C2C12 myogenic differentiation by compromising MyoD. J. Cell Biol..

[52] Lee K.S., Smith K., Amieux P.S., Wang E.H. (2008). MBNL3/CHCR prevents myogenic differentiation by inhibiting MyoD-dependent gene transcription. Differentiation.

[53] Amack J.D., Paguio A.P., Mahadevan M.S. (1999). Cis and trans effects of the myotonic dystrophy (DM) mutation in a cell culture model. Hum. Mol. Genet..

[54] Loro E., Rinaldi F., Malena A., Masiero E., Novelli G., Angelini C., Romeo V., Sandri M., Botta A., Vergani L. (2010). Normal myogenesis and increased apoptosis in myotonic dystrophy type-1 muscle cells. Cell Death Differ..

[55] Larsen J., Pettersson O.J., Jakobsen M., Thomsen R., Pedersen C.B., Hertz J.M., Gregersen N., Corydon T.J., Jensen T.G. (2011). Myoblasts generated by lentiviral mediated MyoD transduction of myotonic dystrophy type 1 (DM1) fibroblasts can be used for assays of therapeutic molecules. BMC Res. Notes.

[56] Michel L., Huguet-Lachon A., Gourdon G. (2015). Sense and antisense DMPK RNA foci accumulate in DM1 tissues during development. PLoS ONE.

[57] Amack J., Mahadevan M. (2001). The myotonic dystrophy expanded CUG repeat tract is necessary but not sufficient to disrupt C2C12 myoblast differentiation. Hum. Mol. Genet..

[58] Bachinski L.L., Sirito M., Böhme M., Baggerly K.A., Udd B., Krahe R. (2010). Altered MEF2 isoforms in myotonic dystrophy and other neuromuscular disorders. Muscle Nerve.

[59] Kalsotra A., Singh R.K., Gurha P., Ward A.J., Creighton C.J., Cooper T.A. (2014). The Mef2 Transcription Network Is Disrupted in Myotonic Dystrophy Heart Tissue, Dramatically Altering miRNA and mRNA Expression. Cell Rep..

[60] Trapnell C., Williams B.A., Pertea G., Mortazavi A., Kwan G., van Baren M.J., Salzberg S.L., Wold B.J., Pachter L. (2010). Transcript assembly and quantification by RNA-Seq reveals unannotated transcripts and isoform switching during cell differentiation. Nat. Biotechnol..

[61] Potthoff M.J., Olson E.N. (2007). MEF2: A central regulator of diverse developmental programs. Development.

[62] Holt I., Jacquemin V., Fardaei M., Sewry C.A., Butler-Browne G.S., Furling D., Brook J.D., Morris G.E. (2009). Muscleblind-Like Proteins. Am. J. Pathol..

[63] Batra R., Charizanis K., Manchanda M., Mohan A., Li M., Finn D.J., Goodwin M., Zhang C., Sobczak K., Thornton C.A. (2014). Loss of MBNL Leads to Disruption of Developmentally Regulated Alternative Polyadenylation in RNA-Mediated Disease. Mol. Cell.

[64] Matloka M., Klein A.F., Rau F., Furling D. (2018). Cells of Matter-In Vitro Models for Myotonic Dystrophy. Front. Neurol..

[65] Faustino N.A., Cooper T.A., Andre N. (2003). Pre-mRNA splicing and human disease. Genes Dev..

[66] Hino S.-I., Kondo S., Sekiya H., Saito A., Kanemoto S., Murakami T., Chihara K., Aoki Y., Nakamori M., Takahashi M.P. (2007). Molecular mechanisms responsible for aberrant splicing of SERCA1 in myotonic dystrophy type. Hum. Mol. Genet..

[67] Fugier C., Klein A.F., Hammer C., Vassilopoulos S., Ivarsson Y., Toussaint A., Tosch V., Vignaud A., Ferry A., Messaddeq N. (2011). Misregulated alternative splicing of BIN1 is associated with T tubule alterations and muscle weakness in myotonic dystrophy. Nat. Med..

[68] Rau F., Lainé J., Ramanoudjame L., Ferry A., Arandel L., Delalande O., Jollet A., Dingli F., Lee K.-Y., Peccate C. (2015). Abnormal splicing switch of DMD’s penultimate exon compromises muscle fibre maintenance in myotonic dystrophy. Nat. Commun..

[69] Wang E.T., Cody N.A.L., Jog S., Biancolella M., Wang T.T., Treacy D.J., Luo S., Schroth G.P., Housman D.E., Reddy S. (2012). Transcriptome-wide regulation of pre-mRNA splicing and mRNA localization by muscleblind proteins. Cell.

[70] Wang E.T., Treacy D., Eichinger K., Struck A., Estabrook J., Olafson H., Wang T.T., Bhatt K., Westbrook T., Sedehizadeh S. (2019). Transcriptome alterations in myotonic dystrophy skeletal muscle and heart. Hum. Mol. Genet..

[71] Richards R.I., Robertson S.A., Kastner D.L. (2018). Neurodegenerative diseases have genetic hallmarks of autoinflammatory disease. Hum. Mol. Genet..

[72] Nakamori M., Hamanaka K., Thomas J.D., Wang E.T., Hayashi Y.K., Takahashi M.P., Swanson M.S., Nishino I., Mochizuki H. (2017). Aberrant Myokine Signaling in Congenital Myotonic Dystrophy. Cell Rep..

[73] Knight J.D., Kothary R. (2011). The myogenic kinome: Protein kinases critical to mammalian skeletal myogenesis. Skelet. Muscle.

[74] Abmayr S.M., Pavlath G.K. (2012). Myoblast fusion: Lessons from flies and mice. Development.

[75] Du H., Cline M.S., Osborne R.J., Tuttle D.L., Clark T.A., Donohue J.P., Hall M.P., Shiue L., Swanson M.S., Thornton C.A. (2010). Aberrant alternative splicing and extracellular matrix gene expression in mouse models of myotonic dystrophy. Nat. Struct. Mol. Biol..

[76] Jun D.Y., Kim H., Jang W.Y., Lee J.Y., Fukui K., Kim Y.H. (2017). Ectopic overexpression of LAPTM5 results in lysosomal targeting and induces Mcl-1 down-regulation, Bak activation, and mitochondria-dependent apoptosis in human HeLa cells. PLoS ONE.

[77] Hsu Y.-C., Perin M.S. (1995). Human Neuronal Pentraxin II (NPTX2): Conservation, Genomic Structure, and Chromosomal Localization. Genomics.

[78] Batra R., Nelles D.A., Krach F., Thomas J.D., Snjader L., Blue S.M., Aigner S., Swanson M.S., Yeo G.W. (2017). Reversal of molecular pathology by RNA-targeting Cas9 in a myotonic dystrophy mouse model. bioRxiv.

[79] Summermatter S., Bouzan A., Pierrel E., Melly S., Fryer C., Leighton-davies J., Glass D.J. (2017). Blockade of metallothioneins 1 and 2 increases skeletal muscle mass and strength. Mol. Cell. Biol..

[80] Urso M.L., Scrimgeour A.G., Chen Y.-W., Thompson P.D., Clarkson P.M. (2006). Analysis of human skeletal muscle after 48 h immobilization reveals alterations in mRNA and protein for extracellular matrix components. J. Appl. Physiol..

[81] Lecker S.H., Jagoe R.T., Gilber A., Gomes M., Baracos V., Bailey J., Price S.R., Mtich W.E., Goldberg A.L. (2004). Multiple types of skeletal muscle atrophy involve a common program of changes in gene expression. FASEB J..

[82] Latres E., Amini A.R., Amini A.A., Griffiths J., Martin F.J., Wei Y., Hsin C.L., Yancopoulos G.D., Glass D.J. (2005). Insulin-like growth factor-1 (IGF-1) inversely regulates atrophy-induced genes via the phosphatidylinositol 3-kinase/Akt/mammalian target of rapamycin (PI3K/Akt/mTOR) pathway. J. Biol. Chem..

[83] Schindelin J., Arganda-Carreras I., Frise E., Kaynig V., Longair M., Pietzsch T., Preibisch S., Rueden C., Saalfeld S., Schmid B. (2012). Fiji: An open-source platform for biological-image analysis. Nat. Methods.

[84] Shen S., Park J.W., Lu Z., Lin L., Henry M.D., Wu Y.N., Zhou Q., Xing Y. (2014). rMATS: Robust and flexible detection of differential alternative splicing from replicate RNA-Seq data. Proc. Natl. Acad. Sci. USA.

[85] Li B., Dewey C.N. (2011). RSEM: Accurate transcript quantification from RNA-Seq data with or without a reference genome. BMC Bioinform..

[86] Langmead B., Salzberg S.L. (2012). Fast gapped-read alignment with Bowtie. Nat. Methods.

[87] Robinson J.T., Thorvaldsdóttir H., Winckler W., Guttman M., Lander E.S., Getz G., Mesirov J.P. (2011). Integrative genomics viewer. Nat. Biotechnol..

[88] Sherry S.T., Ward M.H., Kholodov M., Baker J., Phan L., Smigielski E.M., Sirotkin K. (2001). dbSNP: The NCBI database of genetic variation. Nucleic Acids Res..

[89] Eden E., Navon R., Steinfeld I., Lipson D., Yakhini Z. (2009). GOrilla: A tool for discovery and visualization of enriched GO terms in ranked gene lists. BMC Bioinform..

[90] Chong J., Soufan O., Li C., Caraus I., Li S., Bourque G., Wishart D.S., Xia J. (2018). MetaboAnalyst 4.0: Towards more transparent and integrative metabolomics analysis. Nucleic Acids Res..

