# Supplementary material for: Recovery in the Myogenic Program of Congenital Myotonic Dystrophy Myoblasts after Excision of the Expanded (CTG)n Repeat
Source: Int J Mol Sci. 2019 Nov 13;20(22):5685. doi: 10.3390/ijms20225685 (PMC6888582; doi:10.3390/ijms20225685)
Supplement: Supplementary file 1 [file ijms-20-05685-s001.zip › ijms-642631-supplymentary-proof/Suppl Mat.pdf]

Supplementary Materials (André et al.)

Supplementary Figures

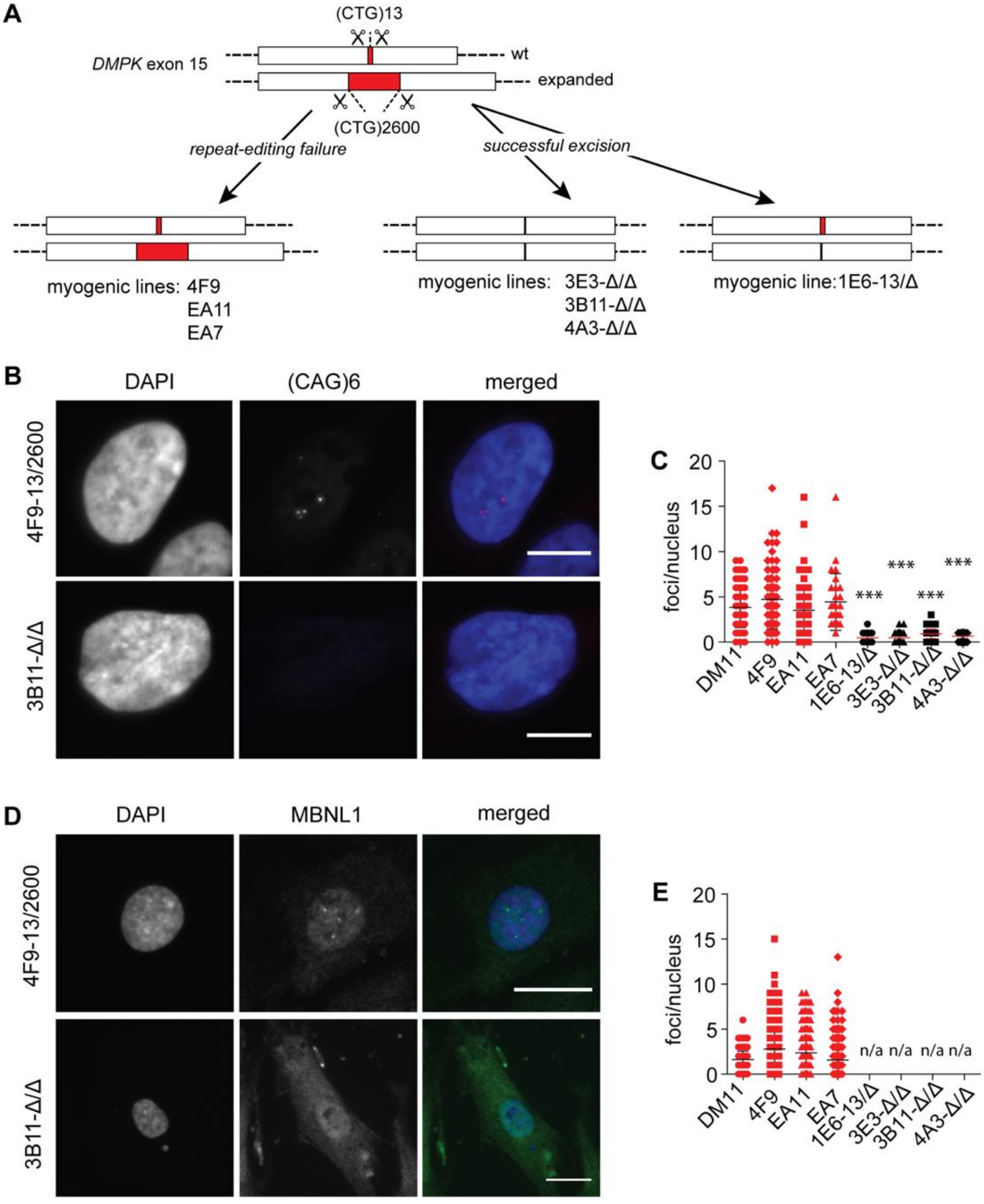

Figure S1. CRISPR/Cas9-mediated excision of the *DMPK* (CTG)2600 repeat results in loss of intranuclear RNA foci and MBNL1 aggregates in cDM myoblasts. (A) Schematic outline of the CRISPR/Cas9 editing procedure by which the panel of isogenic myoblast clones used in this study was obtained. The panel consists of four cell lines with (CTG)13 and (CTG)2600 alleles, i.e. the parental cell line itself originating from an 11-year old female patient with DM1 (Mamchaoui et al., 2011; Arandel et al., 2017) and three non-modified clonal derivatives (4F9, EA11, EA7); one edited clone that had retained only the normal (CTG)13 tract (1E6), and three clones that had both the normal and expanded repeat tracts fully removed (3E3, 3B11, 4A3), i.e. the CTG sequence and an additional 11 bps upstream and 51 bps downstream (Van Agtmaal et al., 2017). (B) Images of *DMPK* (CUG)2600 ribonuclear foci using FISH analysis with the (CAG)6 LNA probe (red). Representative pictures of intranuclear RNA foci in one myoblast line with and one line without the (CTG)2600 repeat are shown. Nuclei were stained with DAPI (blue). Bar = 100 µm. (C) Quantification of RNA foci in the myoblast lines with and without repeat. Each symbol represents the number of foci in one nucleus (mean ± SEM; n = 28-318 (average 110) nuclei per cell line). ***$p<0.001$ (one-way ANOVA). (D) Representative images of MBNL1 immunofluorescence analyses on 4F9-13/2600 and 3B11-Δ/Δ cell lines. Bar = 20 µm (E) Quantification of nuclear MBNL1 foci in repeat-containing cell lines (mean ± SEM, one-way ANOVA). n/a = not analyzed.

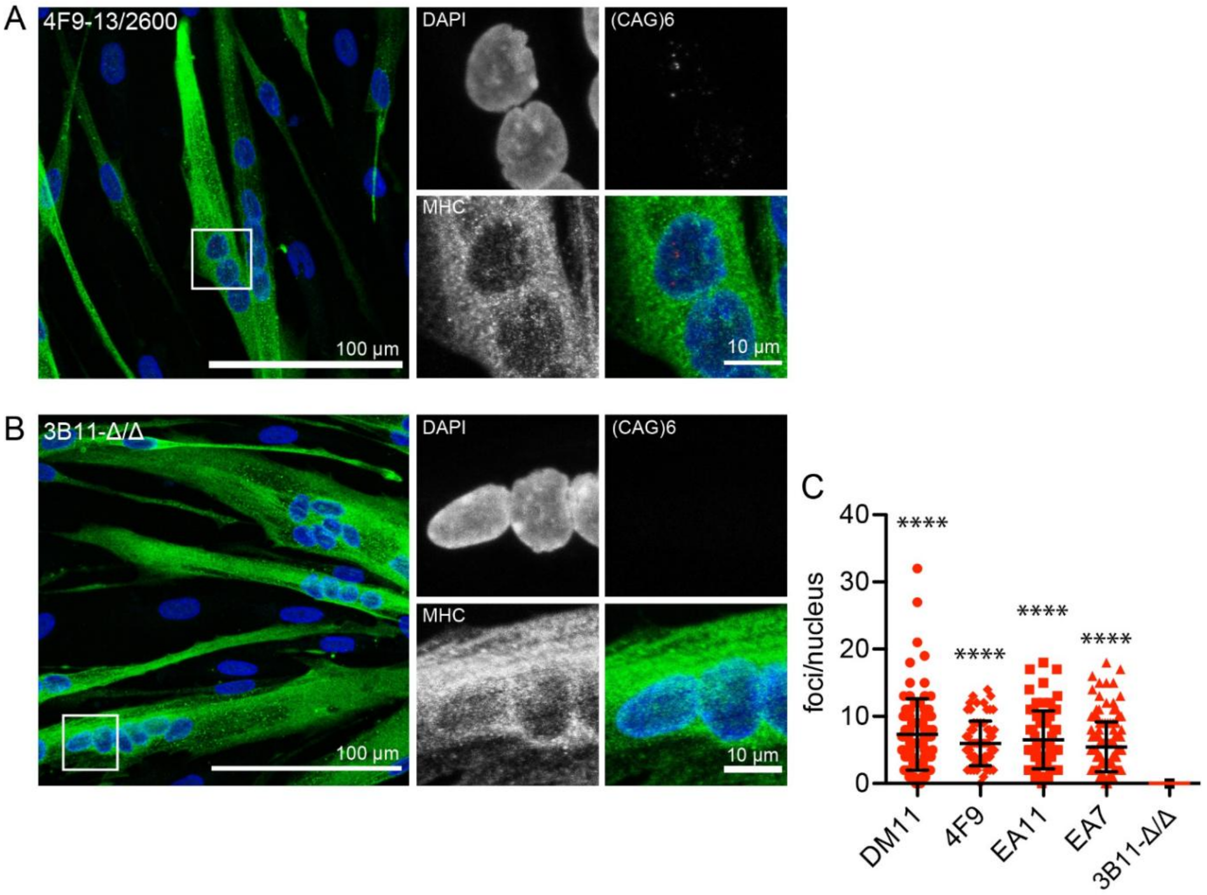

Figure S2. CRISPR/Cas9-mediated excision of the *DMPK* (CTG)2600 repeat results in loss of intranuclear RNA foci in cDM myotubes. Images of *DMPK* (CUG)2600 ribonuclear foci using FISH analysis with the (CAG)6 LNA probe (red). Representative images of intranuclear RNA foci in **(A)** one myogenic cell line with and **(B)** one line without repeat are shown. MHC staining in green and DAPI in blue. White squares indicate the magnified region shown on the right. **(C)** Quantification of RNA foci in myotubes of four lines with and one line without repeat. Each symbol represents the number of foci in one nucleus (mean ± SEM; n=104, 113, 70, 147, and 64 nuclei, respectively). ****p<0.001 (2-way ANOVA).

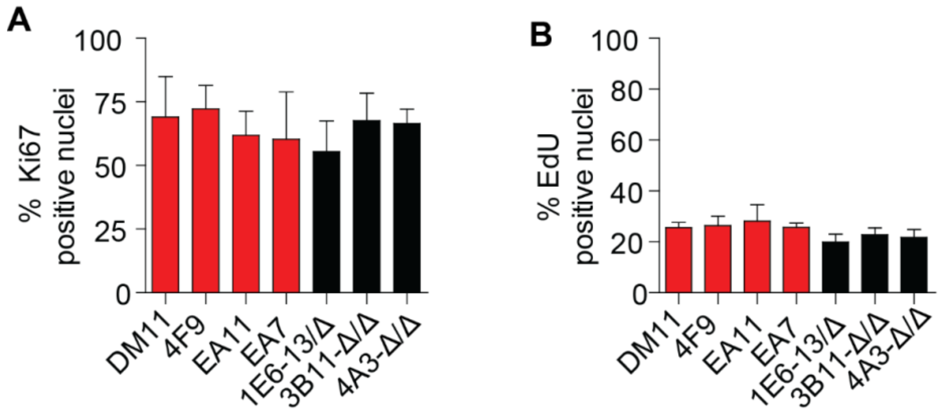

Figure S3. Proliferation capacity of myoblasts with and without expanded (CTG)2600 repeat. **(A)** Myoblasts with Ki67-positive nuclei during non-confluent *in vitro* culture, at day -2 before the onset of myogenic differentiation (mean ± SEM, n=3) **(B)** Myoblasts in S-phase during a one-hour EdU incorporation in non-confluent cell culture (mean ± SEM, n=3).

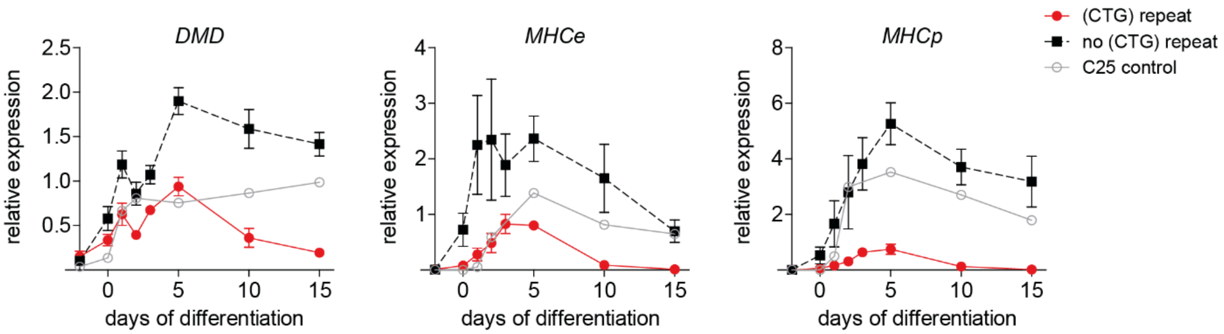

Figure S4. Excision of the (CTG)2600 repeat alters the temporal expression of myogenic progression markers to an expression pattern similar to the expression of an unaffected control cell line. RNA expression for structural muscle proteins *DMD, MHCp* and *MHCe.* Each data point shows mean ± SEM of the four different cell lines either with or without the (CTG)2600 repeat expansion, and non-isogenic control line C25 (See also Fig. 5G-I).

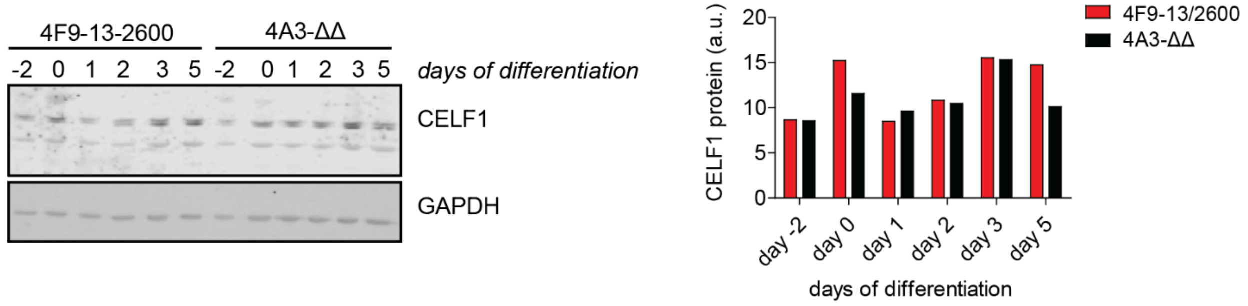

Figure S5. CELF1 protein expression during myogenesis. Western blot images of representative cell lines with (4F9-13/2600) and without (4A3-ΔΔ) the (CTG)2600 repeat (left). Quantification of the western blot signals, using GAPDH as loading control, is shown at the right.

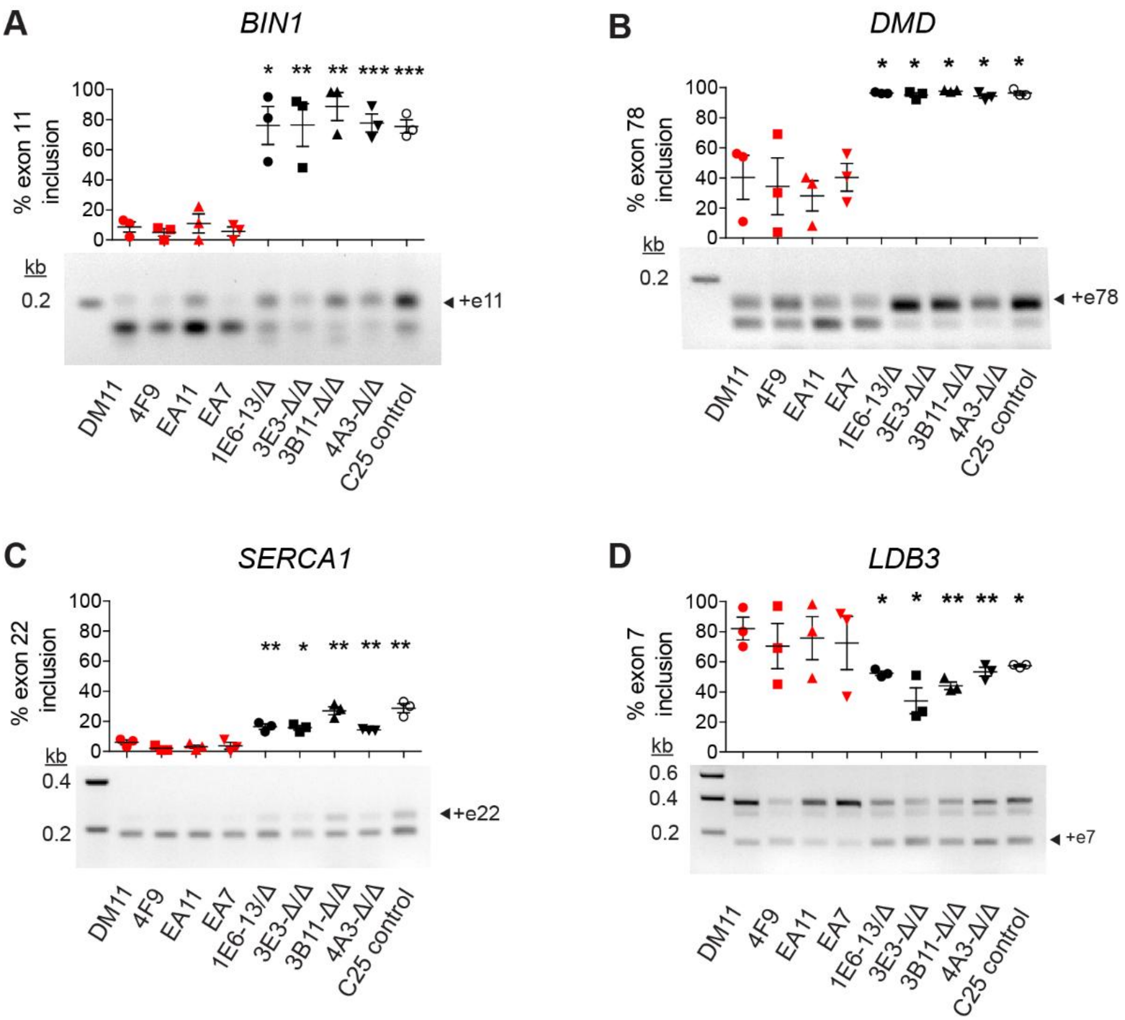

Figure S6. Comparative RT-PCR analysis of *BIN-1*, *DMD*, *SERCA-1* and *LDB3* splicing in cells with and without (CTG)2600 repeat, and non-isogenic control line C25, after five days of differentiation. Note that typical embryonic splicing patterns were reverted to the normal adult modes of splicing in lines without (CTG)2600 repeat expansion, similar to those in the control line. *p<0.05, **p<0.01, ***p<0.001.

Background information on DEGs and DEPs listed in Table S1 and Table S2.

DEGs in Table S1:

XIRP2 (Xin Actin Binding Repeat Containing 2): Exclusively expressed in striated muscle cells, believed to play an important role in development. Xin (encoded by *XIRP1*) and XIRP2 proteins are concentrated at attachment sites of myofibrils. Xin and XIRP2 protein interactions are important during the process during which myofibril are build or rebuild [1,2].

FRG1 (FSHD Muscular Dystrophy Region Gene 1): In C2C12 cell lines overexpression of *FRG1* showed a myoblast fusion defect upon differentiation. Also myoblast isolated from mice overexpressing *FRG1* (showing an FSHD muscular dystrophy phenotype) showed a fusion defect [3]. *FRG1* has been suggested as an epigenetic regulator of muscle differentiation [4].

NGFR (Nerve Growth Factor Receptor): Cell surface receptor that is enriched on hPSC-SMPCs (human pluripotent stem cell – skeletal muscle progenitor cells). Cells positive for NGFR (and ERBB3) were the most myogenic and able to form myotubes more efficiently in vitro [5]. Furthermore, NGFR overexpression in C2C12 leads to enhanced muscle differentiation [6].

TIE1 (Tyrosine kinase with Immunoglobulin-like and EGF-like domains 1): Cognate receptor of Angiopoietin 1. *Tie1* mRNA increases during muscle cell differentiation *in vitro* [7].

IGFBP5 (Insulin-like Growth Factor Binding Protein 5): Promotes skeletal muscle development [8] and reduction of IGFBP5 (along with myogenin and IGF1) is associated with myotube loss and reduced myogenic differentiation in C2C12 cells [9].

ADRA1D (Adrenoceptor Alpha 1D): *ADRA1D* is associated with myotube survival and reduction in ADRA1D levels are associated with reduced myogenic differentiation in C2C12 cells [9].

SOCS2 (Suppressor Of Cytokine Signaling 2): A signaling molecule for the NFkB cascade [10] that interferes with myotube formation through upregulation of *JunB* in C2C12 cells [11].

RUNX1T1 (RUNX1 Translocation Partner 1): RUNX1T1 plays a role in stem cell differentiation as low levels of *RUNX1T1* are detected in undifferentiated ESCs and a gradual increase is observed during differentiation [12].

CYFIP2 (Cytoplasmic FMR Interacting Protein 2): CYFIP2 expression is reduced in mesenchymal stem cells derived from ALS patients compared to healthy control subjects [13].

FILIP1L (Filamin A Interacting Protein 1 Like): Knockdown of *Filip1* inhibits myogenic differentiation in C2C12 cells [14].

FLRT2 (Fibronectin Leucine-Rich Transmembrane Protein 2): FLRT2 is expressed in heart, skeletal muscle and pancreas. Acts as FGF regulator together with other *FLRTs* by interacting with FGFR1 during (mouse) embryogenesis [15].

EDN1 (Endothelin 1): EDN1 impairs insulin-stimulated glucose uptake and was found to be the key gene with an increased expression in C2C12 cells after treatment with a strong differentiation inhibitor (GDF8) [16].

GLUL (Glutamate-Ammonia Ligase): *GLUL* is also known as *glutamine synthetase* (*GS*) and was previously shown to be upregulated in various conditions causing muscle atrophy, usually accompanied by an increase in *FOXO1* expression. An increase in expression of the latter gene was not detected in repeat-containing cells [17,18].

LAPTM5 (Lysosomal Transmembrane 5): *LAPTM5* is highly expressed in haematopoeitic tissues and only lowly in muscle, whose protein product is post-translationally modified, has a role in embryonic development and cell-cycle and apoptosis regulation via involvement in endosomal-lysosomal transport and autophagy in various cell types. A role for this protein - together with its partner CD53 - in efficient formation of myofibers in regenerating muscle at the level of cell fusion has been suggested [2,19].

ABCG2 (ATP Binding Cassette Subfamily G Member 2): *ABCG2* is a gene that participates in muscle regeneration and can be a source of satellite cell replenishment [20,21].

PDE3A (Phosphodiesterase 3A): PDE3A together with its isoform PDE3B is involved in regulation of Ca^{2+} channel physiology and apoptosis of myocytes [22,23].

BMPR1B (Bone Morphogenetic Protein Receptor Type 1B): *BMPR1B* is a protein with a role in BMP signaling, a process with a known role in the inhibition of early myogenesis [2,24].

CASP1 (Caspase 1): Caspases have a role in proteolysis control, as an essential regulatory process in early myogenesis [25–27].

MMP23B (Matrix Metallopeptidase 23B): MMP23B is a gene expressed in prenatal skeletal muscle [28,29].

DCN (Decorin): DCN may be involved in normal muscle differentiation during embryonic myogenesis via regulation of myoblast density by control of migration [2,30–33].

DEPs in Table S2:

MT2A (Metallothionein-2): Silencing of *MT1* and *MT2* results in activation of the *Akt* pathway and increases myotube size in type IIb fiber hypertrophy and increases muscle strength [34].

MBNL (Muscleblind-Like Protein 1): CUG/CCUG expansion transcripts sequester MBNL1 protein in DM1 and DM2 and lower MBNL1 protein levels are reported in DM1. Overexpression of MBNL1 in mouse models rescues myopathy and myotonia [35,36].

CA3 (Carbonic Anhydrase 3): Elevated *CA3* levels are found in blood from Duchenne and Becker muscular dystrophy patients [37], but *CA3* was among the downregulated genes in FSHD patients [2].

AGL (Glycogen Debranching Enzyme): AGL is among the important players in energy metabolism and is significantly down-regulated in ALS muscle tissue [38]. *AGL* is upregulated during myogenesis [39].

FABP5 (Fatty Acid-Binding Protein 5): Along with *FABP4*, *FABP5* has a crucial role in nutrient homeostasis during prolonged fasting and exercise [40] and is downregulated during FSHD myogenesis [2].

MYOZ2 (Myozenin-2): MYOZ2 is a z-disk protein that specifically binds calcineurin [41]. *MYOZ2* is involved in hypertrophic cardiomyopathy [42]. Loss of MYOZ2 and MYOZ1 leads to pronounced loss of type-II muscle fibers in double knock-out mice [43]. MEF2A is bound to MYOZ2, and is thought to regulate myoblast differentiation via regulating MYOZ2 [44].

SP100 (Nuclear Autoantigen Sp-100): SP100 is a transcriptional regulator. Its expression levels are found to be decreased in patients with ischemic cardiomyopathy [45].

TIMP1 (Metalloproteinase Inhibitor 1): Epigenetic reprogramming of *TIMP1* plays a role in smooth muscle responses to mechanical signals [46]. When cardiac function decreases, *TIMP1* levels are decreased as well and are thought to be involved in myocardial remodeling [47].

NCAM1 (Neural Cell Adhesion Molecule 1): NCAM1 levels are increased in neuromuscular junction denervation in age related denervation and loss of skeletal muscle mass and function [48].

COL12A1 (Collagen Alpha-1(XII) chain): Loss-of-function mutations in *COL12A1* underly a muscle and connective tissue disorder [5,49,50].

ITGA4 (Integrin Alpha-4): ITGA4 is a target gene of the *let-7* family members, which are highly dysregulated in *mdm* mouse diaphragm. Overexpression of *let-7* family members leads to decreased expression of *ITGA4* (among other targets) [51].

SDC3 (Syndecan-3): SDC3 is a transmembrane proteoglycan expressed in developing skeletal muscle tissue and on activated satellite cells, suggesting a role in muscle development, homeostasis and regeneration [52–54]. *SDC3* null mice show improved myogenesis and improved muscle ageing [55].

CDK6 (Cyclin-Dependent Kinase 6): CDK6, CDK2 and CDK4 are the G1 CDK enzymes. In C2C12 cells CDK6 activity is low in both proliferating myoblasts as differentiated myotubes, while CDK2 and CDK4 activity decreases during differentiation [56,57].

SDC2 (Syndecan-2): SDC2 is a surface marker on *PAX7*-induced myogenic progenitors, which allows for isolation of myogenic progenitor cells [58].

TPM2 (Tropomyosin Beta Chain): TPM2 is predominantly expressed in skeletal muscle, both in type I as in type II muscle fibers. Mutations in this gene have been linked to different forms of congenital myopathies [59,60].

HMGN1 (Non-Histone Chromosomal Protein HMG-14): HMGN1 binds to chromatin without DNA sequence specificity [61]. While early reports showed how HMGN1 can prevent myogenesis when ectopically expressed in myoblasts [62] an *HMGN1*-null mouse model later showed no muscle differentiation problems [61]. HMGN1 protein shows a gradual decline during myogenic differentiation as a consequence of cell cycle arrest.

NID2 (Nidogen-2): Inhibition of *NID1* and *NID2* interferes with basement membrane stabilization. In *NID1* knock-out mice, *NID2* is upregulated as compensation mechanism in heart and muscle [63]. However, in C2C12 *NID1* declines dramatically during myogenesis while *NID2* remained stable [64].

ITGA11 (Integrin Alpha-11): ITGA11 is a collagen receptor. Its mRNA and protein levels are upregulated during myogenic differentiation in skeletal muscle [65,66].

S100A4 (Protein S100-A4): S100A4 induces proliferation and migration of vascular smooth muscle cells [67] and is among the genes upregulated during FSHD myogenesis [2].

TNNT2 (Troponin T, Cardiac Muscle): TNNT2 is primarily located in cardiac muscle [68], embryonic TNNT2 isoforms re-expression in DM1 adult heart muscle is associated with diminished cardiac efficiency [69].

Supplementary References (including references for Table S1-S3)

1. Eulitz, S.; Sauer, F.; Pelissier, M.-C.; Boisguerin, P.; Molt, S.; Schuld, J.; Orfanos, Z.; Kley, R.A.; Volkmer, R.; Wilmanns, M.; et al. Identification of Xin-repeat proteins as novel ligands of the SH3 domains of nebulin and nebulette and analysis of their interaction during myofibril formation and remodeling. *Mol. Biol. Cell* **2013**, *24*, 3215–3226.
2. Banerji, C.R.S.; Panamarova, M.; Pruller, J.; Figeac, N.; Hebaishi, H.; Fidanis, E.; Saxena, A.; Contet, J.; Sacconi, S.; Severini, S.; et al. Dynamic transcriptomic analysis reveals suppression of PGC1α/ERRα drives perturbed myogenesis in facioscapulohumeral muscular dystrophy. *Hum. Mol. Genet.* **2019**, *28*, 1244–1259.
3. Feeney, S.J.; McGrath, M.J.; Sriratana, A.; Gehrig, S.M.; Lynch, G.S.; D'Arcy, C.E.; Price, J.T.; McLean, C.A.; Tupler, R.; Mitchell, C.A. FHL1 reduces dystrophy in transgenic mice overexpressing FSHD muscular dystrophy region gene 1 (FRG1). *PLoS ONE* **2015**, *10*, e0117665.
4. Neguembor, M.V.; Xynos, A.; Onorati, M.C.; Caccia, R.; Bortolanza, S.; Godio, C.; Pistoni, M.; Corona, D.F.; Schotta, G.; Gabellini, D. FSHD muscular dystrophy region gene 1 binds Suv4-20h1 histone methyltransferase and impairs myogenesis. *J Mol Cell Biol* **2013**, *5*, 294–307.
5. Hicks, D.; Farsani, G.T.; Laval, S.; Collins, J.; Sarkozy, A.; Martoni, E.; Shah, A.; Zou, Y.; Koch, M.; Bönnemann, C.G.; et al. Mutations in the collagen XII gene define a new form of extracellular matrix-related myopathy. *Hum. Mol. Genet.* **2014**, *23*, 2353–2363.
6. Seidl, K.; Erck, C.; Buchberger, A. Evidence for the participation of nerve growth factor and its low-affinity receptor (p75NTR) in the regulation of the myogenic program. *J. Cell. Physiol.* **1998**, *176*, 10–21.
7. McClung, J.M.; Reinardy, J.L.; Mueller, S.B.; McCord, T.J.; Kontos, C.D.; Brown, D.A.; Hussain, S.N.A.; Schmidt, C.A.; Ryan, T.E.; Green, T.D. Muscle cell derived angiopoietin-1 contributes to both myogenesis and angiogenesis in the ischemic environment. *Front. Physiol.* **2015**, *6*.
8. Barthold, J.S.; McCahan, S.M.; Singh, A.V.; Knudsen, T.B.; Si, X.; Campion, L.; Akins, R.E. Altered expression of muscle- and cytoskeleton-related genes in a rat strain with inherited cryptorchidism. *J. Androl.* **2008**, *29*, 352–366.
9. Girven, M.; Dugdale, H.F.; Owens, D.J.; Hughes, D.C.; Stewart, C.E.; Sharples, A.P. l-glutamine Improves Skeletal Muscle Cell Differentiation and Prevents Myotube Atrophy After Cytokine (TNF-α) Stress Via Reduced p38 MAPK Signal Transduction. *J. Cell. Physiol.* **2016**, *231*, 2720–2732.
10. Buford, T.W.; Cooke, M.B.; Willoughby, D.S. Resistance exercise-induced changes of inflammatory gene expression within human skeletal muscle. *Eur. J. Appl. Physiol.* **2009**, *107*, 463–471.
11. Ouyang, X.; Fujimoto, M.; Nakagawa, R.; Serada, S.; Tanaka, T.; Nomura, S.; Kawase, I.; Kishimoto, T.; Naka, T. SOCS-2 interferes with myotube formation and potentiates osteoblast differentiation through upregulation of JunB in C2C12 cells. *J. Cell. Physiol.* **2006**, *207*, 428–436.

12. Jiao, Y.; Huang, B.; Chen, Y.; Hong, G.; Xu, J.; Hu, C.; Wang, C. Integrated Analyses Reveal Overexpressed Notch1 Promoting Porcine Satellite Cells' Proliferation through Regulating the Cell Cycle. *Int J Mol Sci* **2018**, *19*.
13. Nachmany, H.; Wald, S.; Abekasis, M.; Bulvik, S.; Weil, M. Two Potential Biomarkers Identified in Mesenchymal Stem Cells and Leukocytes of Patients with Sporadic Amyotrophic lateral Sclerosis Available online: https://www.hindawi.com/journals/dm/2012/824692/abs/ (accessed on Aug 19, 2019).
14. Militello, G.; Hosen, M.R.; Ponomareva, Y.; Gellert, P.; Weirick, T.; John, D.; Hindi, S.M.; Mamchaoui, K.; Mouly, V.; Döring, C.; et al. A novel long non-coding RNA Myolinc regulates myogenesis through TDP-43 and Filip1. *J Mol Cell Biol* **2018**, *10*, 102–117.
15. Haines, B.P.; Wheldon, L.M.; Summerbell, D.; Heath, J.K.; Rigby, P.W.J. Regulated expression of FLRT genes implies a functional role in the regulation of FGF signalling during mouse development. *Dev. Biol.* **2006**, *297*, 14–25.
16. Wicik, Z.; Sadkowski, T.; Jank, M.; Motyl, T. The transcriptomic signature of myostatin inhibitory influence on the differentiation of mouse C2C12 myoblasts. *Pol J Vet Sci* **2011**, *14*, 643–652.
17. Lecker, S.H.; Jagoe, R.T.; Gilber, A.; Gomes, M.; Baracos, V.; Bailey, J.; Price, S.R.; Mtich, W.E.; Goldberg, A.L. Multiple types of skeletal muscle atrophy involve a common program of changes in gene expression. *The FASEB Journal* **2004**, *18*, 39–51.
18. Kamei, Y.; Hattori, M.; Hatazawa, Y.; Kasahara, T.; Kanou, M.; Kanai, S.; Yuan, X.; Suganami, T.; Lamers, W.H.; Kitamura, T.; et al. FOXO1 activates glutamine synthetase gene in mouse skeletal muscles through a region downstream of 3′-UTR: possible contribution to ammonia detoxification. *American Journal of Physiology-Endocrinology and Metabolism* **2014**, *307*, E485–E493.
19. Jun, D.Y.; Kim, H.; Jang, W.Y.; Lee, J.Y.; Fukui, K.; Kim, Y.H. Ectopic overexpression of LAPTM5 results in lysosomal targeting and induces Mcl-1 down-regulation, Bak activation, and mitochondria-dependent apoptosis in human HeLa cells. *PLoS ONE* **2017**, *12*, e0176544.
20. Doyle, M.J.; Zhou, S.; Tanaka, K.K.; Pisconti, A.; Farina, N.H.; Sorrentino, B.P.; Olwin, B.B. Abcg2 labels multiple cell types in skeletal muscle and participates in muscle regeneration. *Journal of Cell Biology* **2011**, *195*, 147–163.
21. Tamaki, T.; Akatsuka, A.; Ando, K.; Nakamura, Y.; Matsuzawa, H.; Hotta, T.; Roy, R.R.; Edgerton, V.R. Identification of myogenic-endothelial progenitor cells in the interstitial spaces of skeletal muscle. *Journal of Cell Biology* **2002**, *157*, 571–577.
22. Fischmeister, R.; Castro, L.; Abi-Gerges, A.; Rochais, F.; Vandecasteele, G. Species- and tissue-dependent effects of NO and cyclic GMP on cardiac ion channels. *Comp. Biochem. Physiol., Part A Mol. Integr. Physiol.* **2005**, *142*, 136–143.
23. Liu, H.; Maurice, D.H. Expression of cyclic GMP-inhibited phosphodiesterases 3A and 3B (PDE3A and PDE3B) in rat tissues: differential subcellular localization and regulated expression by cyclic AMP. *Br. J. Pharmacol.* **1998**, *125*, 1501–1510.
24. Sartori, R.; Gregorevic, P.; Sandri, M. TGFβ and BMP signaling in skeletal muscle: potential significance for muscle-related disease. *Trends Endocrinol. Metab.* **2014**, *25*, 464–471.
25. Bell, R.A.V.; Al-Khalaf, M.; Megeney, L.A. The beneficial role of proteolysis in skeletal muscle growth and stress adaptation. *Skelet Muscle* **2016**, *6*, 16.
26. Boonstra, K.; Bloemberg, D.; Quadrilatero, J. Caspase-2 is required for skeletal muscle differentiation and myogenesis. *Biochim Biophys Acta Mol Cell Res* **2018**, *1865*, 95–104.
27. Barnoy, S.; Kosower, N.S. Caspase-1-induced calpastatin degradation in myoblast differentiation and fusion: cross-talk between the caspase and calpain systems. *FEBS Lett.* **2003**, *546*, 213–217.
28. Zhao, S.; Zhao, Y.; Niu, P.; Wang, N.; Tang, Z.; Zan, L.; Li, K. Molecular characterization of porcine MMP19 and MMP23B genes and its association with immune traits. *International Journal of Biological Sciences* **2011**, *7*, 1101–1113.

29. Zhao, S.; Zhao, Y.; Niu, P.; Wang, N.; Tang, Z.; Zan, L.; Li, K. Molecular characterization of porcine MMP19 and MMP23B genes and its association with immune traits. *Int. J. Biol. Sci.* **2011**, *7*, 1101–1113.
30. Olguin, H.C.; Santander, C.; Brandan, E. Inhibition of myoblast migration via decorin expression is critical for normal skeletal muscle differentiation. *Dev. Biol.* **2003**, *259*, 209–224.
31. Nishimura, T.; Oyama, K.; Kishioka, Y.; Wakamatsu, J.; Hattori, A. Spatiotemporal expression of decorin and myostatin during rat skeletal muscle development. *Biochem. Biophys. Res. Commun.* **2007**, *361*, 896–902.
32. Riquelme, C.; Larrain, J.; Schonherr, E.; Henriquez, J.P.; Kresse, H.; Brandan, E. Antisense inhibition of decorin expression in myoblasts decreases cell responsiveness to transforming growth factor beta and accelerates skeletal muscle differentiation. *J. Biol. Chem.* **2001**, *276*, 3589–3596.
33. Li, Y.; Li, J.; Zhu, J.; Sun, B.; Branca, M.; Tang, Y.; Foster, W.; Xiao, X.; Huard, J. Decorin gene transfer promotes muscle cell differentiation and muscle regeneration. *Mol. Ther.* **2007**, *15*, 1616–1622.
34. Summermatter, S.; Bouzan, A.; Pierrel, E.; Melly, S.; Stauffer, D.; Gutzwiller, S.; Nolin, E.; Dornelas, C.; Fryer, C.; Leighton-Davies, J.; et al. Blockade of Metallothioneins 1 and 2 Increases Skeletal Muscle Mass and Strength. *Mol Cell Biol* **2017**, *37*.
35. Thomas, J.D.; Sznajder, Ł.J.; Bardhi, O.; Aslam, F.N.; Anastasiadis, Z.P.; Scotti, M.M.; Nishino, I.; Nakamori, M.; Wang, E.T.; Swanson, M.S. Disrupted prenatal RNA processing and myogenesis in congenital myotonic dystrophy. *Genes Dev.* **2017**, *31*, 1122–1133.
36. Chamberlain, C.M.; Ranum, L.P.W. Mouse model of muscleblind-like 1 overexpression: skeletal muscle effects and therapeutic promise. *Hum. Mol. Genet.* **2012**, *21*, 4645–4654.
37. Ayoglu, B.; Chaouch, A.; Lochmüller, H.; Politano, L.; Bertini, E.; Spitali, P.; Hiller, M.; Niks, E.H.; Gualandi, F.; Pontén, F.; et al. Affinity proteomics within rare diseases: a BIO-NMD study for blood biomarkers of muscular dystrophies. *EMBO Mol Med* **2014**, *6*, 918–936.
38. Elf, K.; Shevchenko, G.; Nygren, I.; Larsson, L.; Bergquist, J.; Askmark, H.; Artemenko, K. Alterations in muscle proteome of patients diagnosed with amyotrophic lateral sclerosis. *J Proteomics* **2014**, *108*, 55–64.
39. DiMauro, S.; Spiegel, R. Progress and problems in muscle glycogenoses. *Acta Myol* **2011**, *30*, 96–102.
40. Iso, T.; Haruyama, H.; Sunaga, H.; Matsui, M.; Matsui, H.; Tanaka, R.; Umbarawan, Y.; Syamsunarno, M.R.A.A.; Yokoyama, T.; Kurabayashi, M. Exercise endurance capacity is markedly reduced due to impaired energy homeostasis during prolonged fasting in FABP4/5 deficient mice. *BMC Physiol.* **2019**, *19*, 1.
41. Aurino, S.; Piluso, G.; Saccone, V.; Cacciottolo, M.; D’Amico, F.; Dionisi, M.; Totaro, A.; Belsito, A.; Di Vicino, U.; Nigro, V. Candidate-gene testing for orphan limb-girdle muscular dystrophies. *Acta Myol* **2008**, *27*, 90–97.
42. Osio, A.; Tan, L.; Chen, S.N.; Lombardi, R.; Nagueh, S.F.; Shete, S.; Roberts, R.; Willerson, J.T.; Marian, A.J. Myozenin 2 is a novel gene for human hypertrophic cardiomyopathy. *Circ. Res.* **2007**, *100*, 766–768.
43. Moriscot, A.S.; Baptista, I.L.; Bogomolovas, J.; Witt, C.; Hirner, S.; Granzier, H.; Labeit, S. MuRF1 is a muscle fiber-type II associated factor and together with MuRF2 regulates type-II fiber trophicity and maintenance. *Journal of Structural Biology* **2010**, *170*, 344–353.
44. Wang, Y.-N.; Yang, W.-C.; Li, P.-W.; Wang, H.-B.; Zhang, Y.-Y.; Zan, L.-S. Myocyte enhancer factor 2A promotes proliferation and its inhibition attenuates myogenic differentiation via myozenin 2 in bovine skeletal muscle myoblast. *PLoS ONE* **2018**, *13*, e0196255.
45. Herrer, I.; Roselló-Lletí, E.; Ortega, A.; Tarazón, E.; Molina-Navarro, M.M.; Triviño, J.C.; Martínez-Dolz, L.; Almenar, L.; Lago, F.; Sánchez-Lázaro, I.; et al. Gene expression network analysis reveals new transcriptional regulators as novel factors in human ischemic cardiomyopathy. *BMC Med Genomics* **2015**, *8*, 14.
46. da Silva, R.A.; Fernandes, C.J. da C.; Feltran, G. da S.; Gomes, A.M.; de Camargo Andrade, A.F.; Andia, D.C.; Peppelenbosch, M.P.; Zambuzzi, W.F. Laminar shear stress-provoked cytoskeletal changes are

mediated by epigenetic reprogramming of TIMP1 in human primary smooth muscle cells. *J. Cell. Physiol.* **2019**, *234*, 6382–6396.
47. Wang, X.; Yang, L.; Qi, F.; et al. Myocardial matrix metalloproteinase-3 and matrix metalloproteinase inhibitor-1 expression in congestive heart failure. *Zhonghua Nei Ke Za Zhi* **2002**, *41*, 453–455.
48. Barns, M.; Gondro, C.; Tellam, R.L.; Radley-Crabb, H.G.; Grounds, M.D.; Shavlakadze, T. Molecular analyses provide insight into mechanisms underlying sarcopenia and myofibre denervation in old skeletal muscles of mice. *The International Journal of Biochemistry & Cell Biology* **2014**, *53*, 174–185.
49. Zou, Y.; Zwolanek, D.; Izu, Y.; Gandhy, S.; Schreiber, G.; Brockmann, K.; Devoto, M.; Tian, Z.; Hu, Y.; Veit, G.; et al. Recessive and dominant mutations in COL12A1 cause a novel EDS/myopathy overlap syndrome in humans and mice. *Hum. Mol. Genet.* **2014**, *23*, 2339–2352.
50. Punetha, J.; Kesari, A.; Hoffman, E.P.; Gos, M.; Kamińska, A.; Kostera-Pruszczyk, A.; Hausmanowa-Petrusewicz, I.; Hu, Y.; Zou, Y.; Bönnemann, C.G.; et al. Novel Col12A1 variant expands the clinical picture of congenital myopathies with extracellular matrix defects. *Muscle Nerve* **2017**, *55*, 277–281.
51. Mohamed, J.S.; Hajira, A.; Lopez, M.A.; Boriek, A.M. Genome-wide Mechanosensitive MicroRNA (MechanomiR) Screen Uncovers Dysregulation of Their Regulatory Networks in the mdm Mouse Model of Muscular Dystrophy. *J. Biol. Chem.* **2015**, *290*, 24986–25011.
52. Cornelison, D.D.; Filla, M.S.; Stanley, H.M.; Rapraeger, A.C.; Olwin, B.B. Syndecan-3 and syndecan-4 specifically mark skeletal muscle satellite cells and are implicated in satellite cell maintenance and muscle regeneration. *Dev. Biol.* **2001**, *239*, 79–94.
53. Cornelison, D.D.W.; Wilcox-Adelman, S.A.; Goetinck, P.F.; Rauvala, H.; Rapraeger, A.C.; Olwin, B.B. Essential and separable roles for Syndecan-3 and Syndecan-4 in skeletal muscle development and regeneration. *Genes Dev.* **2004**, *18*, 2231–2236.
54. Pisconti, A.; Cornelison, D.D.W.; Olguín, H.C.; Antwine, T.L.; Olwin, B.B. Syndecan-3 and Notch cooperate in regulating adult myogenesis. *J. Cell Biol.* **2010**, *190*, 427–441.
55. Pisconti, A.; Banks, G.B.; Babaeijandaghi, F.; Betta, N.D.; Rossi, F.M.V.; Chamberlain, J.S.; Olwin, B.B. Loss of niche-satellite cell interactions in syndecan-3 null mice alters muscle progenitor cell homeostasis improving muscle regeneration. *Skelet Muscle* **2016**, *6*, 34.
56. Franklin, D.S.; Xiong, Y. Induction of p18INK4c and its predominant association with CDK4 and CDK6 during myogenic differentiation. *Mol. Biol. Cell* **1996**, *7*, 1587–1599.
57. Chikenji, A.; Ando, H.; Nariyama, M.; Suga, T.; Iida, R.; Gomi, K. MyoD is regulated by the miR-29a-Tet1 pathway in C2C12 myoblast cells. *J Oral Sci* **2016**, *58*, 219–229.
58. Magli, A.; Incitti, T.; Kiley, J.; Swanson, S.A.; Darabi, R.; Rinaldi, F.; Selvaraj, S.; Yamamoto, A.; Tolar, J.; Yuan, C.; et al. PAX7 Targets, CD54, Integrin α9β1, and SDC2, Allow Isolation of Human ESC/iPSC-Derived Myogenic Progenitors. *Cell Rep* **2017**, *19*, 2867–2877.
59. Citirak, G.; Witting, N.; Duno, M.; Werlauff, U.; Petri, H.; Vissing, J. Frequency and phenotype of patients carrying TPM2 and TPM3 gene mutations in a cohort of 94 patients with congenital myopathy. *Neuromuscul. Disord.* **2014**, *24*, 325–330.
60. Borovikov, Y.S.; Rysev, N.A.; Karpicheva, O.E.; Sirenko, V.V.; Avrova, S.V.; Piers, A.; Redwood, C.S. Molecular mechanisms of dysfunction of muscle fibres associated with Glu139 deletion in TPM2 gene. *Sci Rep* **2017**, *7*, 16797.
61. Hill, D.A.; Imbalzano, A.N. HMGN1 is dispensable for myogenesis and adipogenesis. *Gene* **2006**, *371*, 59–67.
62. Pash, J.M.; Alfonso, P.J.; Bustin, M. Aberrant expression of high mobility group chromosomal protein 14 affects cellular differentiation. *J. Biol. Chem.* **1993**, *268*, 13632–13638.
63. Miosge, N.; Sasaki, T.; Timpl, R. Evidence of nidogen-2 compensation for nidogen-1 deficiency in transgenic mice. *Matrix Biol.* **2002**, *21*, 611–621.
64. Neu, R.; Adams, S.; Munz, B. Differential expression of entactin-1/nidogen-1 and entactin-2/nidogen-2 in myogenic differentiation. *Differentiation* **2006**, *74*, 573–582.

65. Velling, T.; Kusche-Gullberg, M.; Sejersen, T.; Gullberg, D. cDNA cloning and chromosomal localization of human alpha(11) integrin. A collagen-binding, I domain-containing, beta(1)-associated integrin alpha-chain present in muscle tissues. *J. Biol. Chem.* **1999**, *274*, 25735–25742.
66. Rinella, L.; Marano, F.; Berta, L.; Bosco, O.; Fraccalvieri, M.; Fortunati, N.; Frairia, R.; Catalano, M.G. Extracorporeal shock waves modulate myofibroblast differentiation of adipose-derived stem cells. *Wound Repair Regen* **2016**, *24*, 275–286.
67. Choe, N.; Kwon, D.-H.; Shin, S.; Kim, Y.S.; Kim, Y.-K.; Kim, J.; Ahn, Y.; Eom, G.H.; Kook, H. The microRNA miR-124 inhibits vascular smooth muscle cell proliferation by targeting S100 calcium-binding protein A4 (S100A4). *FEBS Lett.* **2017**, *591*, 1041–1052.
68. Wei, B.; Jin, J.-P. TNNT1, TNNT2, and TNNT3: Isoform genes, regulation, and structure-function relationships. *Gene* **2016**, *582*, 1–13.
69. Choi, J.; Dixon, D.M.; Dansithong, W.; Abdallah, W.F.; Roos, K.P.; Jordan, M.C.; Trac, B.; Lee, H.S.; Comai, L.; Reddy, S. Muscleblind-like 3 deficit results in a spectrum of age-associated pathologies observed in myotonic dystrophy. *Sci Rep* **2016**, *6*, 30999.
70. Fugier, C.; Klein, A.F.; Hammer, C.; Vassilopoulos, S.; Ivarsson, Y.; Toussaint, A.; Tosch, V.; Vignaud, A.; Ferry, A.; Messaddeq, N.; et al. Misregulated alternative splicing of BIN1 is associated with T tubule alterations and muscle weakness in myotonic dystrophy. *Nat. Med.* **2011**, *17*, 720–725.
71. Rau, F.; Lainé, J.; Ramanoudjame, L.; Ferry, A.; Arandel, L.; Delalande, O.; Jollet, A.; Dingli, F.; Lee, K.-Y.; Peccate, C.; et al. Abnormal splicing switch of DMD’s penultimate exon compromises muscle fibre maintenance in myotonic dystrophy. *Nat Commun* **2015**, *6*, 7205.
72. Kimura, T.; Nakamori, M.; Lueck, J.D.; Pouliquin, P.; Aoike, F.; Fujimura, H.; Dirksen, R.T.; Takahashi, M.P.; Dulhunty, A.F.; Sakoda, S. Altered mRNA splicing of the skeletal muscle ryanodine receptor and sarcoplasmic/endoplasmic reticulum Ca2+-ATPase in myotonic dystrophy type 1. *Hum. Mol. Genet.* **2005**, *14*, 2189–2200.
73. François, V.; Klein, A.F.; Beley, C.; Jollet, A.; Lemercier, C.; Garcia, L.; Furling, D. Selective silencing of mutated mRNAs in DM1 by using modified hU7-snRNAs. *Nat. Struct. Mol. Biol.* **2011**, *18*, 85–87.
